# Metagenomic insights into the response of soil microbial communities to pathogenic *Ralstonia solanacearum*

**DOI:** 10.3389/fpls.2024.1325141

**Published:** 2024-02-16

**Authors:** Yansong Xiao, Sai Zhang, Hongguang Li, Kai Teng, Shaolong Wu, Yongbin Liu, Fahui Yu, Zhihong He, Lijuan Li, Liangzhi Li, Delong Meng, Huaqun Yin, Yujie Wang

**Affiliations:** ^1^ Chenzhou Tobacco Company of Hunan Province, Changsha, China; ^2^ Xiangxi Tobacco Co Hunan Prov, Changsha, China; ^3^ Hunan Tobacco Research Institute, Changsha, China; ^4^ School of Minerals Processing and Bioengineering, Central South University, Changsha, China

**Keywords:** soil microbial community, bacterial wilt, metagenomics, interactions, HGT

## Abstract

Understanding the response of soil microbial communities to pathogenic *Ralstonia solanacearum* is crucial for preventing bacterial wilt outbreaks. In this study, we investigated the soil physicochemical and microbial community to assess their impact on the pathogenic *R.solanacearum* through metagenomics. Our results revealed that certain archaeal taxa were the main contributors influencing the health of plants. Additionally, the presence of the pathogen showed a strong negative correlation with soil phosphorus levels, while soil phosphorus was significantly correlated with bacterial and archaeal communities. We found that the network of microbial interactions in healthy plant rhizosphere soils was more complex compared to diseased soils. The diseased soil network had more linkages, particularly related to the pathogen occurrence. Within the network, the family Comamonadaceae, specifically *Ramlibacter_tataouinensis*, was enriched in healthy samples and showed a significantly negative correlation with the pathogen. In terms of archaea, *Halorubrum*, *Halorussus_halophilus* (family: Halobacteriaceae), and *Natronomonas_pharaonis* (family: Haloarculaceae) were enriched in healthy plant rhizosphere soils and showed negative correlations with *R.solanacearum*. These findings suggested that the presence of these archaea may potentially reduce the occurrence of bacterial wilt disease. On the other hand, *Halostagnicola_larseniia* and *Haloterrigena*_sp._BND6 (family: Natrialbaceae) had higher relative abundance in diseased plants and exhibited significantly positive correlations with *R.solanacearum*, indicating their potential contribution to the pathogen’s occurrence. Moreover, we explored the possibility of functional gene sharing among the correlating bacterial pairs within the Molecular Ecological Network. Our analysis revealed 468 entries of horizontal gene transfer (HGT) events, emphasizing the significance of HGT in shaping the adaptive traits of plant-associated bacteria, particularly in relation to host colonization and pathogenicity. Overall, this work revealed key factors, patterns and response mechanisms underlying the rhizosphere soil microbial populations. The findings offer valuable guidance for effectively controlling soil-borne bacterial diseases and developing sustainable agriculture practices.

## 1 Introduction

A growing global population and variety of agricultural pathogens pose a serious danger to food security through crop diseases, yet in many regions of the world, there is insufficient infrastructure to enable prompt diagnosis of these diseases. (Shafi et al., 2017; Ahmed et al., 2022a). Bacterial wilt is a typical soil-borne disease caused by *R.solanacearum* (Jiang et al., 2017). Due to its wide host range and high mortality rate, bacterial wilt has become a major problem, which hinders the development of agricultural industry (Ahmed et al., 2022a).

The pathogen *R.solanacearum* are able to infect over 250 plant species in 54 monocot and dicot botanical families, exhibiting an exceptionally wide host range (Ahmed et al., 2022b). The Solanaceae (e.g., tomato, tobacco and pepper) could be capable to study the pathogen interaction (Peeters et al., 2013). Tobacco is one of the important cash crops in China, the cultivation of tobacco can be threatened by bacterial wilt, and caused enormous economic losses (Wang et al., 2017; Ahmed et al., 2022a). Therefore, limiting and eliminating the occurrence of bacterial wilt has emerged as a crucial component of contemporary agricultural advancement.

There are many methods to prevent and control the incidence of bacterial wilt, including cultural control, chemical control, crop rotation and soil removal (Meline et al., 2023). However, these methods have never achieved satisfactory results in preventing and controlling bacterial wilt invasion (Addy et al., 2012; Wei et al., 2017). Biological control (Ahmed et al., 2022b; Li et al., 2022) options emerge as promising alternatives to assist crops to fight pathogens (Rahman et al., 2018). Successfully microbial biocontrol depends on numerous factors, such as environmental conditions, ecological changes and genetic types (Wang et al., 2017). Effective preventing the incidence of bacterial wilt requires a comprehensive understanding of the pathogenesis of bacterial wilt.

Soil microbiota plays a vital role in regulating plant growth and health (Guo et al., 2022; Dai et al., 2023). Native microorganisms also can inhibit the growth of invasive pathogens through mechanisms such as nutrient competition or material antagonism (Bonanomi et al., 2018; Ahmed et al., 2022a). Certain soils naturally suppress plant pathogens; this indicates that these soils have specific characteristics to control plant pathogens (Deng et al., 2022; Zhang et al., 2022). Microorganisms, including bacteria and fungi, are the main soil suppression drivers. Previous research has demonstrated that rhizosphere bacteria can use material antagonism and nutrient competition to stop the spread of invasive diseases (Yin et al., 2021; Zhong et al., 2022). In addition, disease-suppressing soil also reduces the susceptibility of the next generation plants to pathogens, this phenomenon can be called “soil-borne legacy” (Bakker et al., 2018). And the interactions between microbial species in the soil environment become more complex under the influence of environmental factors (Niu et al., 2020). Understanding the association of microbial communities with disease suppression can provide a basis for soil community regulation, and it also provide new opportunities to promote plant health in a sustainable manner (Zhang et al., 2022). However, the ecological changes induced by these interactions are difficult to elucidate, especially for soils (Wu et al., 2023). The composition, changes and interaction of soil microorganisms in inhibiting bacterial wilt pathogenic bacteria, and the changes in the rhizosphere soil environment during this process, need to be studied.

The regulative effect of microbial interactions plays an instrumental role in biocontrol of soil-borne phytopathogens (Niu et al., 2020; Wu et al., 2023). In this study, the healthy and the diseased (the outbreak of bacterial wilt) plant rhizosphere soils under different growth-stages were investigated by soil physicochemical analysis and metagenomics. The interactions between plant rhizosphere soil microorganisms and the pathogenic *R.solanacearum*, and the changes of soil environment of healthy and diseased plants were comprehensively analyzed by the co-occurrence network. the horizontally transferred genes were identified by the Integrated Microbial Genomes Annotation Pipeline. This study can contribute to ensure the healthy condition of planting soils, and promote the prevention of bacterial wilt disease.

## 2 Materials and methods

### 2.1 Site description and sampling

All samples were collected from Guiyang County (25°75’97”N, 112°74’05”E), which is a subtropical monsoon humid climate located in Chenzhou city of Hunan Province, China. Pei et al. (Pei et al., 2022) indicated that Guiyang was a high-risk region to appeared the meteorological disaster (e.g., hail, floods, high temperature), which could remarkably cause plant disease, and influence the growth and quality of crops. Tobacco as the main crops in this area was in exigent of the prevention of the bacterial wilt invasion.

The experiment object was the tobacco (*Nicotiana* tabacum) of “yunyan87”. The healthy and diseased plants were collected in the high incidence of bacterial wilt disease field. We recognized diseased plant by the characteristics of invasion with bacterial wilt, and categorized plants into healthy (showing no visible symptoms of infection) or diseased (showing visible symptoms of infection) groups. Sampling was performed under three stages in May to July of 2022. The initial stage involved healthy plant samples collected on May 18th (CK1) in boom period of tobacco plants, which generally did not include diseased plants. The invasion of bacterial wilt by tobacco plants had a high incidence of bacterial wilt disease in mature period of tobacco plants. Therefore, we set two sampling time to comprehensively understand the processing of bacterial wilt invasion. The middle and final stages involved collecting healthy (CK2 and CK3) and diseased (R2 and R3) plant samples on June 18th and July 2nd, with a 2-week interval between each sampling. At each stage, the rhizosphere soils were randomly collected using the five-point method for the soil *physicochemical* and microbial analysis. Each of the five sub-samples was taken from a single plant within the study area, merged, and divided into three duplicates. Stones and other debris, as well as plant remnants, were manually removed from all soil samples before being processed. Sterilized Ziplock bags were filled with the collected soils. The rhizosphere soils were sampled using Edwards’ technique (Edwards et al., 2015), and the rice plants in each treatment, together with their rhizosphere soils, were meticulously observed. Samples are then brought to the lab and placed in a portable refrigerator (4 °C). Every soil sample was split into two sections: one was air-dried for physicochemical analysis, and the other was kept at -80 °C for microbial DNA extraction.

### 2.2 Soil physicochemical analysis

The soil samples were transported to the laboratory, where they were allowed to air dry at ambient temperature before being crushed and put through a 2 mm screen. The methods of soil physical and chemical analysis (1978; Yang et al., 2023) were followed in performing the soil physicochemical analysis. In particular, two stages of drying were used to measure the water content of the soil (per 100 g fresh weight). Using a pH meter (PHS-3C, Lei-ci), the soil pH was measured (soil/water = 2.5/1, *w/w*). Potassium dichromate (K_2_Cr_2_O_7_) and sulfuric acid (H_2_SO_4_) oxidation and titration were used to measure the organic matter (OM). An analytical instrument that operates on continuous flow was used to determine the levels of NO3−-N and NH4+-N (San++, Skalar, Holland). Available potassium (AK) was measured using flame photometry, while the amount of available phosphorus (AP) was found using a colorimetric approach. The semimicro Kjeldahl, fused sodium hydroxide-colorimetric, and sodium hydroxide-flame photometry techniques were employed to measure the contents of total nitrogen (TN), phosphorus (TP), and potassium (TK), respectively.

### 2.3 Soil microbial community analysis

#### 2.3.1 DNA extraction and PCR amplification

The E.Z.N.A.® Stool DNA Kit (D4015-02, Omega, Inc., USA) was used to extract DNA. Unused swabs that completed DNA extraction and testing to ensure they had no DNA amplicons were utilized as sample blanks. After being eluted in 50 µl of elution buffer, the whole DNA was preserved at -80 °C. dsDNA Fragmentase (NEB, M0348S) was used to fragment DNA for 30 minutes at 37 °C. With the fragmented cDNA, the TruSeq Nano DNA LT Library Preparation Kit library formation process was started. Exonuclease activity in conjunction with fill-in reactions produces blunt-end DNA fragments. With the sample purification beads that were given, size selection was carried out. Each strand’s blunt ends were given an A-base in order for it to be ligated to the indexed adapters. Due to their T-base overhang, the adapters can ligate to the fragmented DNA with an A tail. Each adapter has all of the sequencing primer hybridization sites required for single, paired-end, and indexed reads in addition to a T-base overhang for ligating the adapter to the A-tailed fragmented DNA. The following conditions were used for PCR amplification of the ligated products after fragments were ligated using single or dual index adapters: eight cycles of denaturation at 98 °C for 15 seconds, annealing at 60 °C for 15 seconds, and extension at 72 °C for 30 seconds; and finally, final extension at 72 °C for 5 minutes. At last, we performed the 2×150bp paired-end sequencing (PE150) on an illumina Novaseq™ 6000 (LC-Bio Technology Co., Ltd., Hangzhou, China) following the vendor’s recommended protocol.

#### 2.3.2 Data processing and taxonomic annotation

Several procedures were taken in order to prepare the raw sequencing reads for further analysis. Using cutadapt v1.9, sequencing adapters were first eliminated (Kechin et al., 2017). Next, a sliding-window method was used by fqtrim v0.94 to trim low-quality readings. After that, bowtie2 (Langmead and Salzberg, 2012) was used to match the reads to the host genome in order to eliminate host contamination. To create the metagenome for each sample, quality-filtered reads were acquired and assembled using SPAdes v3.10.0 (Bankevich et al., 2012). MetaGeneMark v3.26 was used to predict all of the coding regions (CDS) of metagenomic contigs. To obtain unigenes, the CDS sequences of all samples were clustered using CD-HIT v4.6.1 (Li and Godzik, 2006). According to Langmead and Salzberg (2012), TPM evaluated the unigene abundance for a given sample based on the quantity of aligned reads obtained using bowtie2 v2.2.0. We used the standard database for taxonomic categorization of short reads along with Kraken 2 (Wood et al., 2019) to calculate the abundance at the species level. Bracken (Lu et al., 2017) was used to adjust counts and determine the total number of readings categorized. All community analysis and statistics were carried out by microeco (Liu et al., 2021) package in R software (version 4.1.2). Using the Vegan package, PcoA (Principal Coordinate Analysis) based on the Bray-Curtis dissimilarity matrix was used to depict community dissimilarity and ordination plots (Dixon, 2003). We used Linear discriminant analysis effect size (LEfSe) (Segata et al., 2011) to detect substantially distinct taxa across five groups. Analysis of Lefse was done according to phylum, genus, and species.

### 2.4 Co-occurrence network

The co-occurrence correlation network was established in Cytoscape using CoNet (Faust and Raes, 2016). only the samples that occurred in row minimum sum = 0.01 were retained to subsequent statistics. Microbial interaction is revealed by Spearman’s correlation threshold of 0.8 (p<0.05). A Cytoscape software plugin: MCODE (Molecular complex detection) (Bader and Hogue, 2003), was utilized for module analysis. Every node in a network has a size that corresponds to the number of connections (i.e., degree). The Gephi software was used to visualize networks (Jacomy et al., 2014). After MENs (Molecular Ecological Network Analysis) results, we further investigate the Likelihood (ML) of functional gene sharing between correlated bacterial pairs within MENs. In particular, potential HGT events from the corresponding taxa in MENs were found using the genomes of rhizosphere microbial isolates that were available. The Integrated Microbial Genomes Annotation Pipeline (IMGAP) v.5.0 (Markowitz et al., 2010) was used to identify horizontally transferred genes in the genomes of bacterial isolates from rhizosphere. This pipeline defined genes in plant genomes that were tested as having been horizontally transferred from a distant lineage in accordance with the following principle: genes in comparison to lower-scoring hits or no hits within the taxonomic lineage of the examined genome, have the greatest BLASTP hits (highest bit scores) or >90% of the best hits discovered outside the lineage. Using the Maximum LM method and 1,000 bootstrap replicates (with bootstrap values in red showing on respective branches), the phylogeny of various abiotic resistance gene types was constructed based on gene-translating protein sequences using the PhyML program (Guindon et al., 2010). The results were visualized with iTOL (Letunic and Bork, 2021). Before building trees, sequences were aligned using MUSCLE (Edgar, 2004) and trimmed using Gblocks (Talavera and Castresana, 2007).

### 2.5 Statistic analysis

ANOVA was used to determine variations in metabolite concentrations between the five groups. Using the linkET (v 0.0.7.4) package, the Mantel test was performed to find the correlation between microorganisms and the plant rhizosphere soil, with significant differences across the five groups by cor (method = Spearman). Using the Pheatmap (v1.0.12) software, heatmaps were created to visually represent variations in the relative abundances, and Euclidean distances between important fungal species were computed (Murtagh and Legendre, 2014). In order to determine the main statistically important microorganism predictors of pathogenic *R.solanacearm* relative abundance by RandomForest (4.7-1.1), we performed a classification random forest (RF) analysis (tree = 2000). Using Adobe Illustrator CC 2019 (Adobe Systems Inc., San Francisco, CA, United States), all figures were processed and illustrated.

## 3 Results

### 3.1 Soil physicochemical properties

The rate of water content showed a significant increase (*p* < 0.05) in the middle stage of plant growth, with values of 22.3% (CK2) and 25.7% (R2) for healthy and diseased plant samples, respectively. However, this trend was not found in the last stage. The pH levels of diseased plant samples were generally lower than those of healthy plant samples. Especially, the pH of R2 was significantly (*p* < 0.05) lower than CK2. Additionally, the levels of AP, TP and TK showed a decreasing trend in the middle and last stages. Generally, no significant differences were found between OM, AN, AK and TN of five group samples (**Table 1**). These results indicated that the environment of plant rhizosphere was considerably influenced by the occurrence of bacterial wilt disease.

**Table 1:** Comparison of the measured factors for soil samples collected from the plant rhizosphere soils.

	CK1	CK2	R2	CK3	R3
Water content (%)	16.6 ± 0.7c	22.3 ± 1.5b	25.7 ± 2.2a	13.2 ± 1.8d	13.4 ± 1.9d
pH	6.3 ± 0.2ab	7.5 ± 0.3a	5.2 ± 1.2b	6.8 ± 1.7ab	5.1 ± 0.1b
OM (g·kg^-1^)	33.6 ± 4.1a	26.2 ± 4.3a	23.8 ± 14.9a	25.2 ± 4.6a	26.7 ± 8.2a
AN (mg·kg^-1^)	147.7 ± 33.1a	127 ± 14.1a	108.7 ± 50.9a	109.5 ± 18.4a	124.4 ± 38.4a
AP (mg·kg^-1^)	224 ± 86.5a	112.0 ± 29.6b	104.7 ± 20.6b	83.7 ± 15.5b	96.0 ± 5.3b
AK (mg·kg^-1^)	1083.3 ± 406.5a	700.0 ± 100.0a	625 ± 66.1a	733.3 ± 308.6a	883.3 ± 137.7a
TN (g·kg^-1^)	2.0 ± 0.1a	1.7 ± 0.3a	1.5 ± 1.0a	1.5 ± 0.4a	2.0 ± 0.6a
TP (g·kg^-1^)	1.7 ± 0.5a	1.4 ± 0ab	1.1 ± 0.1b	1.0 ± 0.1b	1.1 ± 0.3b
TK (g·kg^-1^)	10.6 ± 1.0ab	9.6 ± 0.4ab	13.9 ± 4.6a	9.6 ± 1.1ab	8.8 ± 1.4b

The same superscript letter indicates no significant (p > 0.05) difference between the means according to one-way ANOVA (Duncan) analysis, and different letters indicate significant (p < 0.05) difference. The data are presented as mean ± standard deviation.

OM, organic matter; AN, available nitrogen; AP, available phosphorus; AK, available kalium; TN, total nitrogen; TP, total phosphorus; TK, total kalium.

### 3.2 Microbial community structure and composition

**Figure 1:**
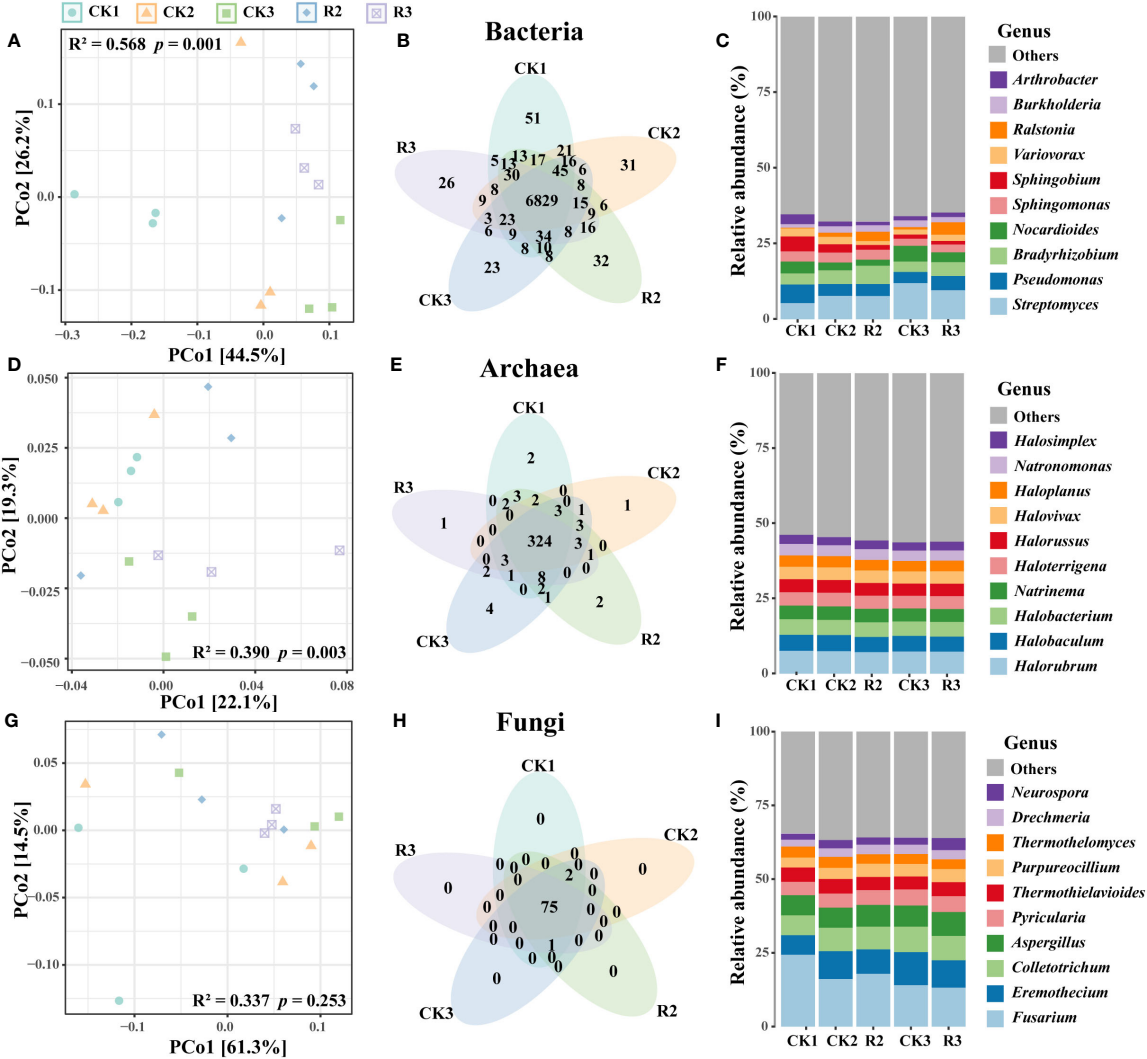
Microbial community structure and composition of the rhizosphere soils in five groups. PcoA showing the Bray-Curtis similarities of bacterial **(A)**, archaeal **(D)** and fungal **(G)** communities. Venn diagrams of bacterial **(B)**, archaeal **(E)** and fungal **(H)** illustrating the numbers of shared and unique OTUs among five groups. The composition of bacterial **(C)**, archaeal **(F)** and fungal **(I)** communities in terms of genus.

PCoA showed that bacterial and archaeal communities were well separated across five groups (*p* < 0.05, adonis test) (**Figures 1A, D**). For fungi, there was no evident change of composition of major species under these five groups (**Figure 1G**). Moreover, the abundance of unique bacterial OTUs (Operational Taxonomic Units) were higher in CK1 than the other four groups, while unique OTUs in archaeal and fungal communities were rare (**Figures 1B, E, H**). These findings indicated that the occurrence of disease influenced the bacterial and archaeal communities, and restricted unique of soil bacterial species.

Bacterial communities were primarily composed of phyla Proteobacteria (58.9%), Actinobacteria (32.5%), Bacteroidetes (2.1%), Firmicutes (1.7%), Planctomycetes (1.2%), Acidobacteria (1.1%) (**Supplementary Figure S1A**). The top five genera *Streptomyces* (8.5%), *Pseudomonas* (4.5%), *Bradyrhizobium* (4.5%), *Nocardioides* (3.4%) were belonged to Proteobacteria and Actinobacteria (**Figure 1C**). The majority of archaea belonged to phyla Euryarchaeota (94.3%), Thaumarchaeota (4.1%) and Crenarchaeota (1.4%) (**Supplementary Figure S1B**). The top five genera within the archaeal community were *Sphingomonas* (3.0%), *Halorubrum* (7.4%), *Halobaculum* (5.2%), *Halobacterium* (5.0%), *Natrinema* (4.4%), *Haloterrigena* (4.4%), all of which were part of Euryarchaeota (**Figure 1F**). As for the fungal communities, they were predominantly composed of the phyla Ascomycota (87.8%) and Basidiomycota (12.1%) (**Supplementary Figure S2C**). The top five genera *Fusarium* (17.1%), *Eremothecium* (9.0%), *Colletotrichum* (7.9%), *Aspergillus* (7.3%), *Pyricularia* (5.0%) were belonged to Ascomycota, and collectively comprising over half of the entire community. Noticeable, *Streptomyces* and *Nocardioides* (12.0% and 5.2%) had the highest relative abundance in CK3, respectively. *Ralstonia* (3.1% and 4.1%) were higher in R2 and R3 than that in healthy plant soils, the pathogenic bacteria *R.solanacearum* (0.3% and 0.4%) were exhibited same trend with *Ralstonia* (**Supplementary Figure S2**) (**Figure 1I**).

### 3.3 Key taxa differentiating healthy and diseased samples

**Figure 2:**
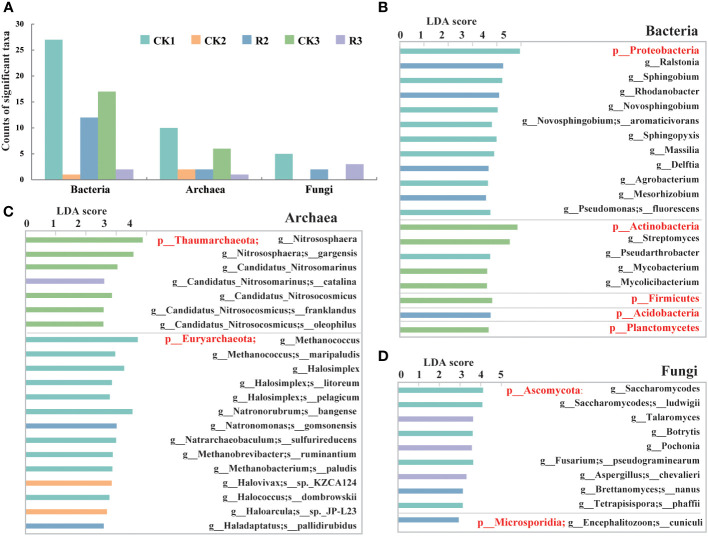
The microbial taxa identified as biomarkers. Bar chart of microbial taxa identified as biomarkers in the rhizosphere soils of five groups by LEfSe (LDA < 2, *p* < 0.05) **(A)**. Bar chart of bacterial (LDA < 3.5, *p* < 0.05) **(B)**, archaeal **(C)** and fungal **(D)** communities (LDA < 2, *p* < 0.05) in terms of phylum, genus and species.

Generally, a total of 59 bacterial taxa were identified as biomarkers (LDA > 3, *p* < 0.05) (**Supplementary Table S1**), and 21 and 10 taxa were identified as biomarkers of archaea and fungi among five groups (LDA > 2, p < 0.05) (**Figure 2A**). The CK1 group exhibited the largest number of enriched taxa, which can serve as biomarkers for distinguishing the taxa that cause bacterial wilt disease with growing time. Specifically, 27, 12 and 17 of 59 key bacterial taxa were found to be abundant in CK1, R2 and CK3, respectively, indicating the remarkable changes in these groups. Among the archaeal, 10 and 6 of 21 significantly (*p*<0.05) different taxa enriched in CK1 and CK3, respectively, suggesting a possible association between these changes and a healthy status of plants. In the terms of fungi, 2 and 3 of 10 fungal taxa were found in R2 and R3, respectively, while no fungal key taxa were found in CK2 and CK3 groups. The results indicated that the variation of certain taxa can have an impact on the health of the samples.

For bacteria, Proteobacteria and most their subclassified genera and species were abundant in CK1, including certain genera (e.g., *Ralstonia*, *Rhodanobacter*, *Delftia*) that were enriched in R2. Actinobacteria and most of their subclassified genera (e.g., *Streptomyces*, *Pseudarthrobacter*, *Mycobacterium*) were enriched in CK1 and CK3, while Acidobacteria and their subclassified genera (e.g., *Granulicella*, *Terriglobus*, *Edaphobacter*) were enriched in R2 (**Figure 2B**, **Supplementary Table S1**). The distribution suggested that the variation of Proteobacteria, Acidobacteria and Actinobacteria could be vital factors contributing to bacterial wilt over time. As for archaea, except for catalina (g_*Candidatus*_Nitrosomarinus) enriched in R3, Thaumarchaeota and their genera and species of CK3 had the highest relative abundance among five groups. Euryarchaeota and their major genera and species were mainly distributed in CK1. *Natronomonas gomsonensis* and *Haladaptatus pallidirubidus* were dominating taxa in R2, while Halovivax sp._KZCA124 (g_*Halovivax*) and Haloarcula sp._JP-L23 (g_*Haloarcula*) were most enriched in CK2 (**Figure 2C**). For fungi, Ascomycota and their subclassified genera and species were primarily distributed in CK1, with consisting of *Talaromyces, Pochonia, chevalieril* (g_*Aspergillus*) had the highest relative abundance in R3. Only one specie, cuniculi (g_*Encephalitozoon*), belonging to the phylum of Microsporidia was enriched in R2 (**Figure 2D**). These findings indicated that the changes observed in these key taxa were considerably correlated to the occurrence of bacterial wilt.

### 3.4 Interaction processes of microbial communities driven by pathogen

**Figure 3:**
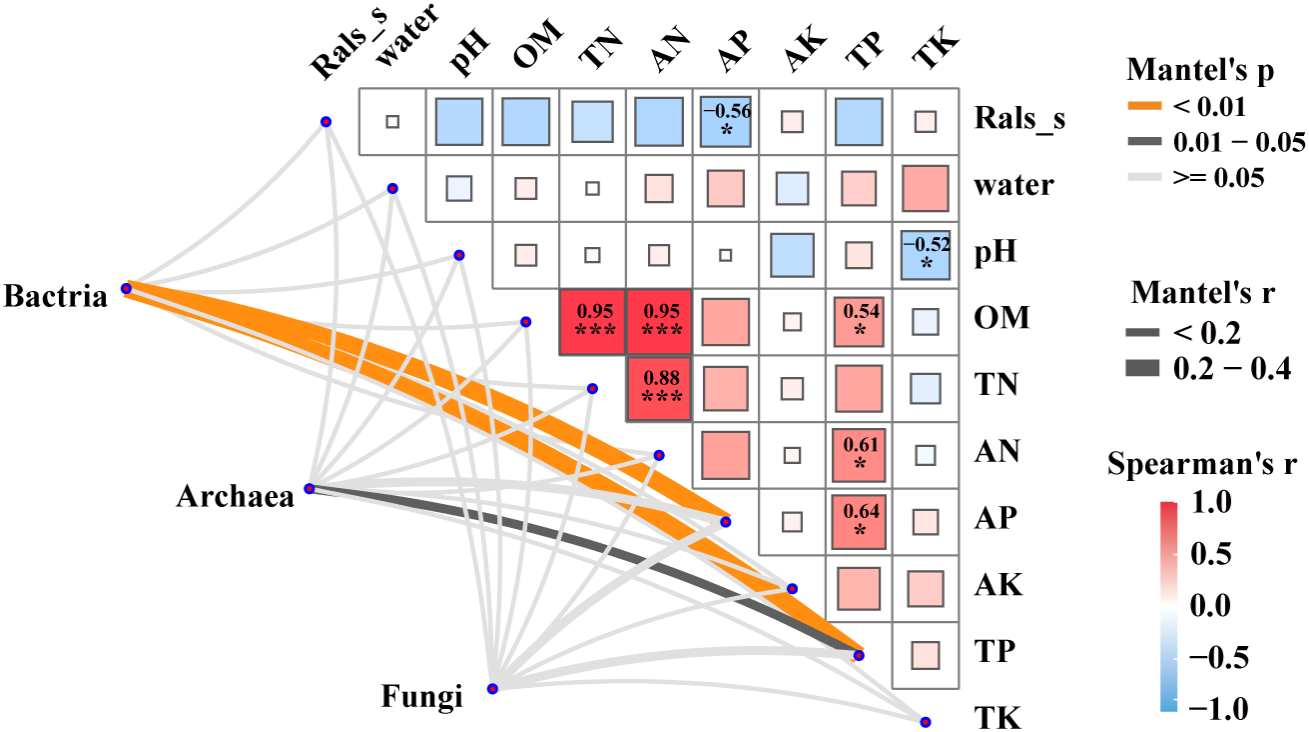
Factors driving microbial communities in five groups. Pairwise Spearman’s correlation matrix of the pathogen *R.solanacearm* and the soil properties of plant rhizosphere was shown with block charts. and microbial (bacterial, archaeal and fungal) communities were related to pathogen and the soil properties by Mantel tests. Edge width means the Mantel’s statistic, and edge color means the statistical significance. *, **, *** represent *P* < 0.05, 0.01, 0.001, respectively.

Mantel tests were performed to reveal the correlation between the microbial communities (bacteria, archaea and fungi), the relative abundance of pathogenic *R. solanacearm* and the soil properties of plant rhizosphere soils (**Figure 3**). The results revealed significant correlations (*p* < 0.05) between bacterial and archaeal communities and soil physicochemical factors, such as AP and TP. Notably, soil AP exhibited a strong negative correlation with the relative abundance of the pathogenic *R.solanacearm.* Additionally, TP showed a strong positive correlation with OM, AN, and AP. These findings highlight the importance of considering the interplay between microbial communities, soil properties, and the occurrence of bacterial wilt. The results revealed significant correlations (*p* < 0.05) between bacterial and archaeal communities and soil physicochemical factors, such as AP and TP. Notably, soil AP exhibited a strong negative correlation with the relative abundance of the pathogen *R.solanacearm*. On the other hand, TP showed a strong positive correlation with organic matter (OM), ammonium nitrogen (AN), and available phosphorus (AP). These findings highlight the importance of considering the interplay between microbial communities, soil properties, and the occurrence of bacterial wilt.

**Supplementary Table S1** displays the topological characteristics of the microbial communities in both healthy and pathological states, as revealed by MEN studies. In comparison to the diseased plant rhizosphere soils network (278 nodes and 2746 edges), the healthy plant rhizosphere soils network (275 nodes and 3197 edges) had more connections, suggesting a reduction in microbial interactions in the diseased plant compared to the healthy plant. Using the average number of neighbors as a proxy for network complexity, it was possible to determine that the rhizosphere soils of healthy plants had a higher level of complexity (23.251 for healthy soils compared to 19.755 for sick soils). The number of edges, characteristic path length, clustering coefficient, density, heterogeneity and centralization in healthy and diseased soils were 3197 and 2746, 2.841 and 2.911, 0.516 and 0.497, 0.085 and 0.071, 0.495 and 0.398, 0.117 and 0.070, respectively. These statistics displayed the usual small world network traits since they differed significantly from the other networks.

**Figure 4:**
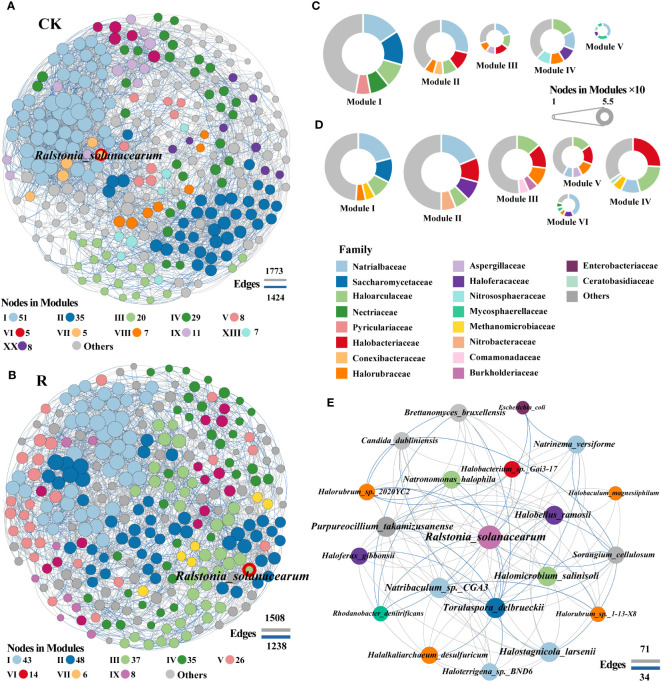
Co-occurrence network patterns of microbial communities of healthy (CK) and diseased (R) samples. The network of healthy **(A)** and disease **(B)** samples. A connection indicates a strong (Spearman’s |r| >0.8) and significant (*p* < 0.05) correlation. Positive correlations are represented by gray lines, whereas negative correlations are represented by blue lines. The size of the nodes corresponds to the degree of taxonomic species. A summary of node-edge statistics was also given, and numbers represent the nodes (in each module) or edges. The pie charts represent the composition of modules with >10 nodes in healthy **(C)** and disease **(D)** co-occurrence network. Network of interactions between the pathogen (red border) and other species in diseased plant rhizosphere soils. The color of pie chart area in **(C, D)** and nodes in **(E)** represented the level of family.

To gain a deeper insight into the interactions among soil microorganisms, the healthy and diseased network modular proportions were visualized and found to exhibit significantly different modular proportions (**Figures 4C, D**). The network modules of healthy and diseased proportions appeared to be significantly altered with the microbial communities. The numbers of module for healthy and diseased soils were 20 and 12, respectively. The pathogenic *R.solanacearm* was in module VII and III of healthy and diseasedwith 5 and 37 linked nodes (**Figures 4A, B**), respectively. The linkages showed that the interactions among potential pathogenic *R.solanacearm* and other microbial members were more in the networks of diseased than healthy soils. Specifically, the top five families Haloarculaceae, Halobacteriaceae, Halorubraceae, Burkholderiaceae and Comamonadaceae were appeared in modular III of diseased plant rhizosphere soils network, which were abundantly found in healthy network. Especially, Burkholderiaceae and Comamonadaceae were also barely found in the other modules of diseased plant rhizosphere soils network. These results suggested that these two families may play vital roles in suppressing the outbreak of bacterial wilt.

Subnetworks for the interactions between pathogenic *R.solanacearm* and other microbial members were analyzed to identify “inferred” key organisms in diseased root rhizosphere soils in order to further reveal which rhizosphere soil microorganisms may be important in assisting or inhibiting bacterial wilt outbreaks (**Figure 4E**). The network of interactions in diseased root rhizosphere soils revealed that the pathogenic bacteria (*R.solanacearm*) were negatively correlated with several genera, including the bacteria of *Sorangium* (family: Polyangiaceae), *Rhodanobacter* (family: Rhodanobacteraceae), *Escherichia* (family: Enterobacteriaceae), the archaea of *Halorubrum* (family: Halorubrum) and the fungi of *Torulaspor*a (family: Torulaspora). Conversely, *R.solanacearm* were positively correlated with archaea, including *Natronomonas* and *Halomicrobium* (2, family: Haloarculaceae), *Halobacterium* (family: Halobacteriaceae), *Halobellus*, *Haloferax*, *Halalkaliarchaeum*, *Halorubrum* and *Halobaculum* (5, family: Halorubraceae), *Halostagnicola*, *Natrinema*, *Natribaculum*, *Haloterrigena* (4, family: Natrialbaceae) and fungi *Purpureocillium* and *Brettanomyces*. These archaea, fungi, and bacteria may be crucial in aiding and preventing bacterial wilt infections since they associated negatively and favorably, respectively, with potentially pathogenic *R.solanacearm*. These findings suggested that during the infection phase of wilt disease, positively correlated archaea that aid in colonization and/or are enriched through a mutualistic interaction in plant roots rhizosphere soils may be the primary factor supporting pathogen invasion.

### 3.5 Changes of specific microorganisms associated with pathogen

**Figure 5:**
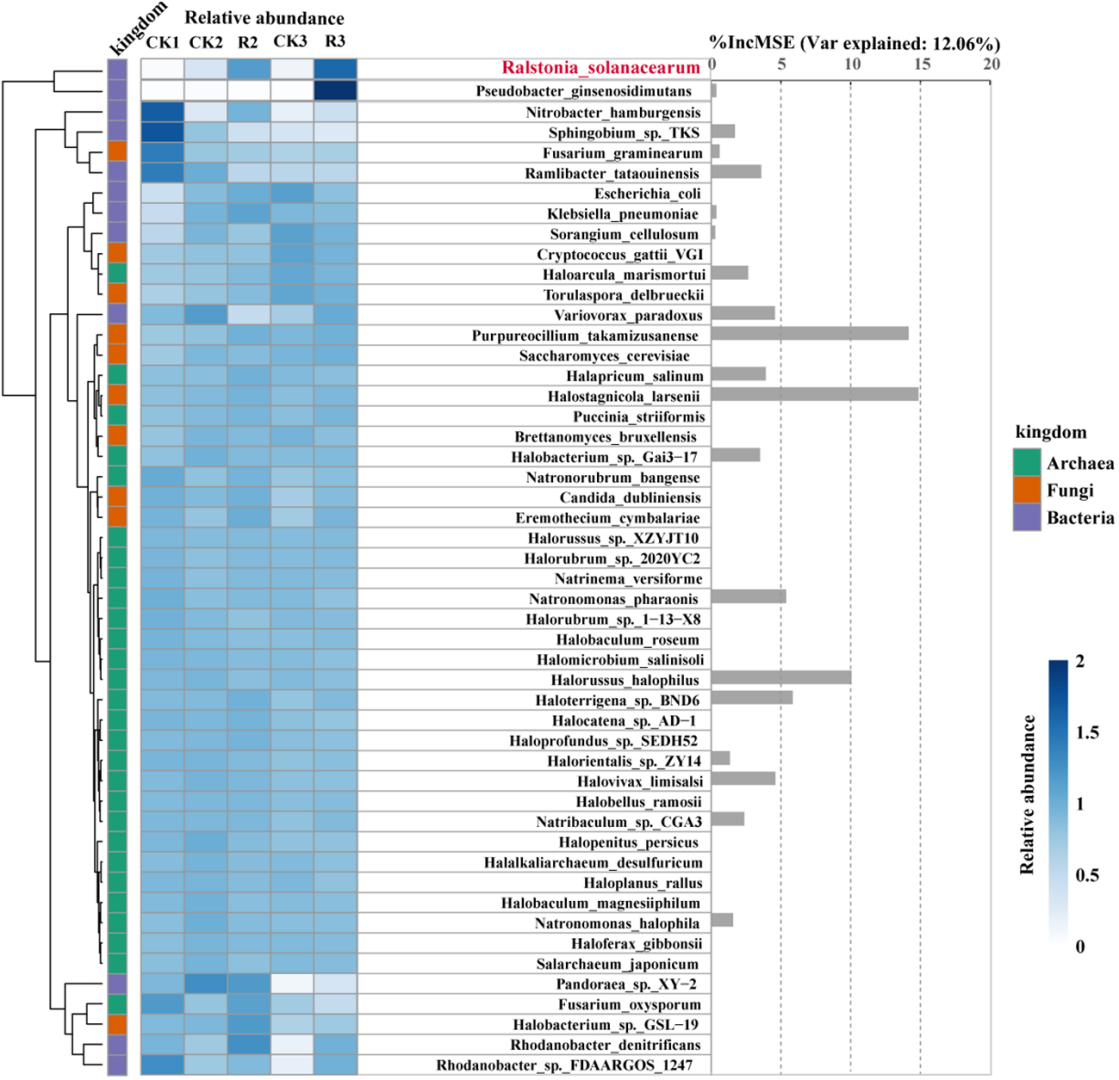
Relative abundance and contribution of specific microorganisms associated with the pathogenic *R.solanacearm*. Heatmap and hierarchical cluster analysis based on the standardized relative abundance (left), while bar diagram (right) based on the random forest contribution. The darker the color, the higher the relative abundance of those microorganisms. In total of 50 microorganisms correlated with *R.solanacearm* and the nodes whereof *R.solanacearm* in modular of diseased soils. These were identified as predictors of *R.solanacearm* relative abundance by random forest analysis. The accuracy importance measure was computed for each tree and averaged over the forest (2000 trees). The percentage increases in the mean squared error (MSE) of variables were used to estimate the importance of these predictors, with higher MSE% values indicating more important predictors.

To explore the changes of specific microorganisms correlated with *R.solanacearm* and the nodes whereof *R.solanacearm* in module of diseased soils. We conducted a random forest analysis of the relative abundance of 49 specific species to reveal their contribution to the occurrence of pathogenic *R.solanacearm*. Generally, heatmap and hierarchical cluster analysis were intuitively reflected the differences of species relative abundance of five groups (**Figure 5**). The contribution of them to the relative abundance of pathogenic *R.solanacearm* was 46.32%. Our findings showed that *Pseudobacter_ginsenosidimutans* (family: Chitinophagaceae) was enriched in R3 with a positive correlation to the pathogen. *Sphingobium*_sp._TKS (family: Sphingomonadaceae) and *Ramlibacter*_*tataouinensis* (family: Comamonadaceae) were enriched in CK1 with significantly negative correlation (*p* < 0.05). *Halostagnicola*_*larsenii*, *Purpureocillium*_*takamizusanense*, *Halorussus*_*halophilus*, *Haloterrigena*_sp._BND6 and *Natronomonas*_*pharaonis* had above 5% increase in mean squared error, but the changes of their relative abundance were variable. *Halostagnicola*_larsenii (family: Natrialbaceae) had higher relative abundance in diseased plants (R2 and R3) than that in the other healthy plants, which had the highest contribution (%IncMSE: 14.86) to *R.solanacearm* among 49 specific species. *Purpureocillium*_takamizusanense (family: Ophiocordycipitaceae) and *Haloterrigena*_sp._BND6 (family: Natrialbaceae) had a highest relative abundance in R2, respectively. Noticeably, Halostagnicola_larsenii, Purpureocillium_takamizusanense and *Haloterrigena*_sp._BND6 had significantly (*p* < 0.05) positive correlations with the pathogen *R.solanacearm*. On the contrary, the relative abundance of *Halorussus*_halophilus (family: Halobacteriaceae) and *Natronomonas*_pharaonis (family: Haloarculaceae) enriched in healthy plant rhizosphere soils with negative correlations to *R.solanacearm*, indicated that the presence of them could potentially reduce the occurrence of bacterial wilt. These findings indicated that *R.solanacearm*, as pathogen, interacting with these specific taxa could contribute to the outbreak of bacterial wilt.

**Figure 6:**
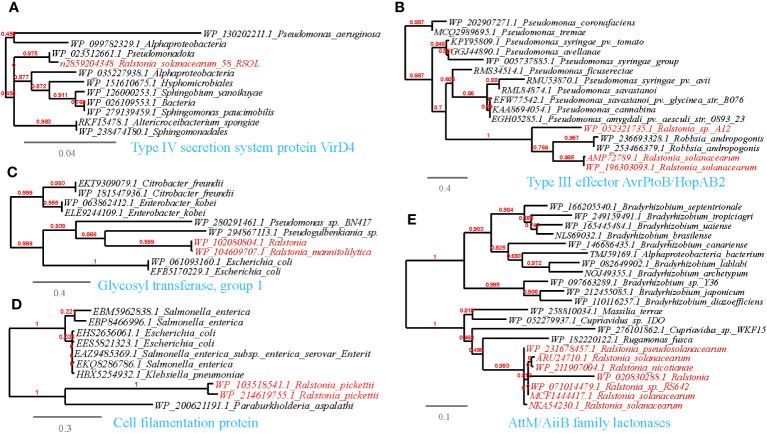
Phylogenetic analyses of the representative HGT genes of *Ralstonia* spp. related with attachment and entry into host tissue in this study (marked with red color) with the existing sequences from Genbank database (constructed with PhyML, the branch length is proportional to the number of substitutions per site): Type IV secretion system protein VirD4 **(A)**, Type III effector AvrPtoB/HopAB2 **(B)**, Glycosyl transferase, group I **(C)**, Cell filamentation protein **(D)**, AltM/AiiB family lactonases **(E)**.

**Figure 7:**
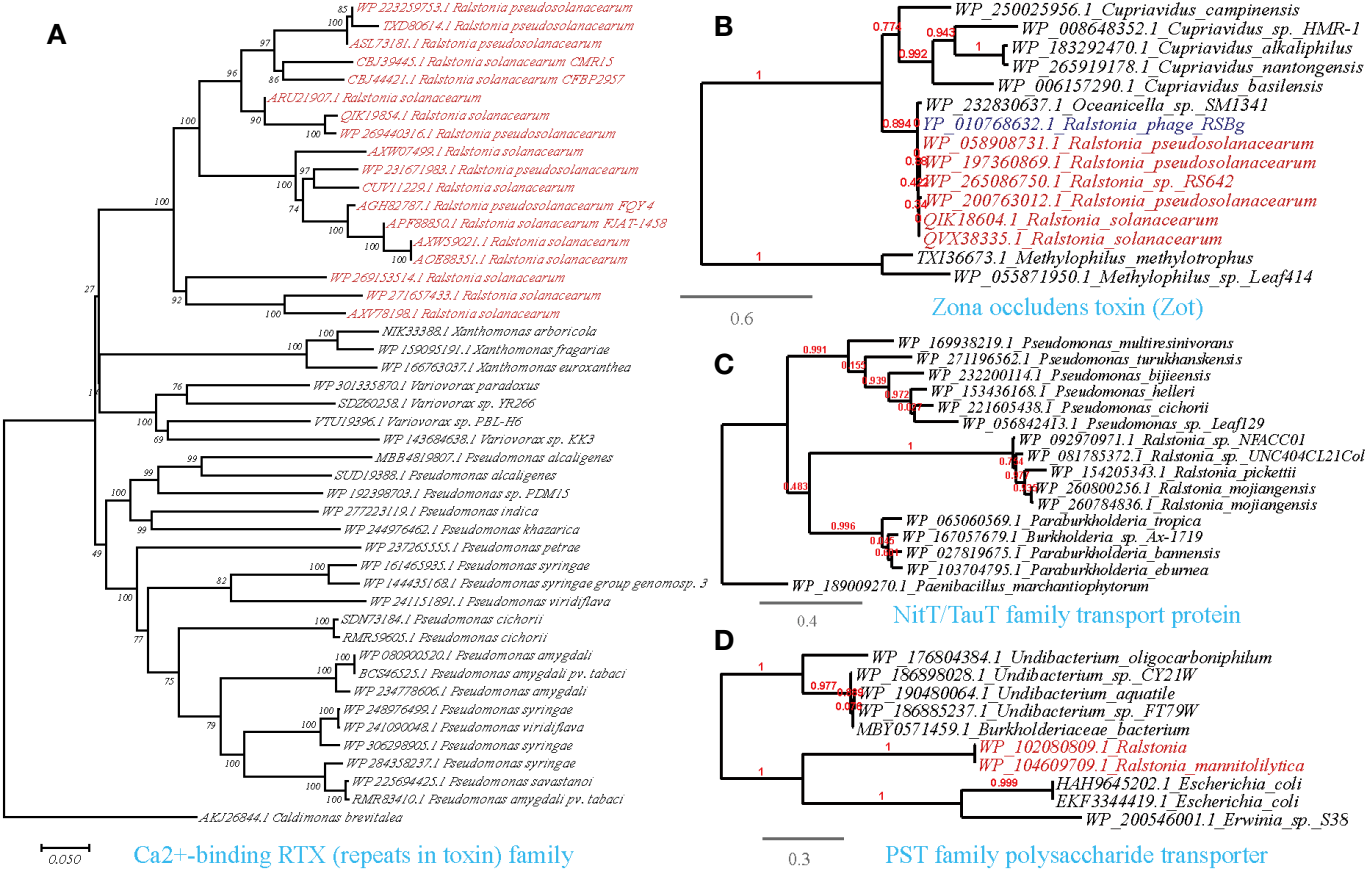
Phylogenetic analyses of the representative HGT genes of *Ralstonia* spp. related with ingestion and utilization of nutrients from the host in this study (marked with red color) with the existing sequences from Genbank database (constructed with PhyML, the branch length is proportional to the number of substitutions per site): Ca^2+^-binding RTX (repeats in toxin) family **(A)**, Zona occludens toxin (Zot) **(B)**, NitT/TauT family transport protein **(C)**, PST family polysaccharide transporter **(D)**.

**Figure 8:**
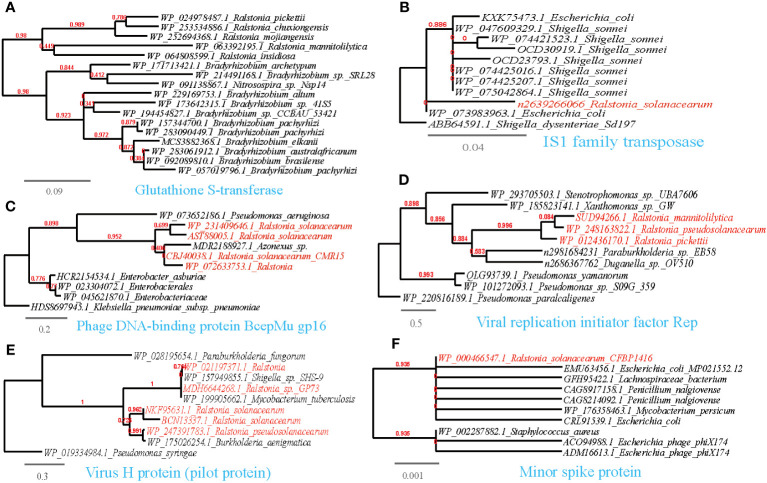
Phylogenetic analyses of the representative HGT genes of *Ralstonia* spp. related with counteraction, subvert, or manipulation of host pathways in this study (marked with red color) with the existing sequences from Genbank database (constructed with PhyML, the branch length is proportional to the number of substitutions per site): Glutathione S-transferase **(A)**, IS1 family transposase **(B)**, Phage DNA-binding protein BcepMu gp16 **(C)**, Viral replication initiator factor Rep **(D)**, Virus H protein (pilot protein) **(E)**, Minor spike protein **(F)**.

**Figure 9:**
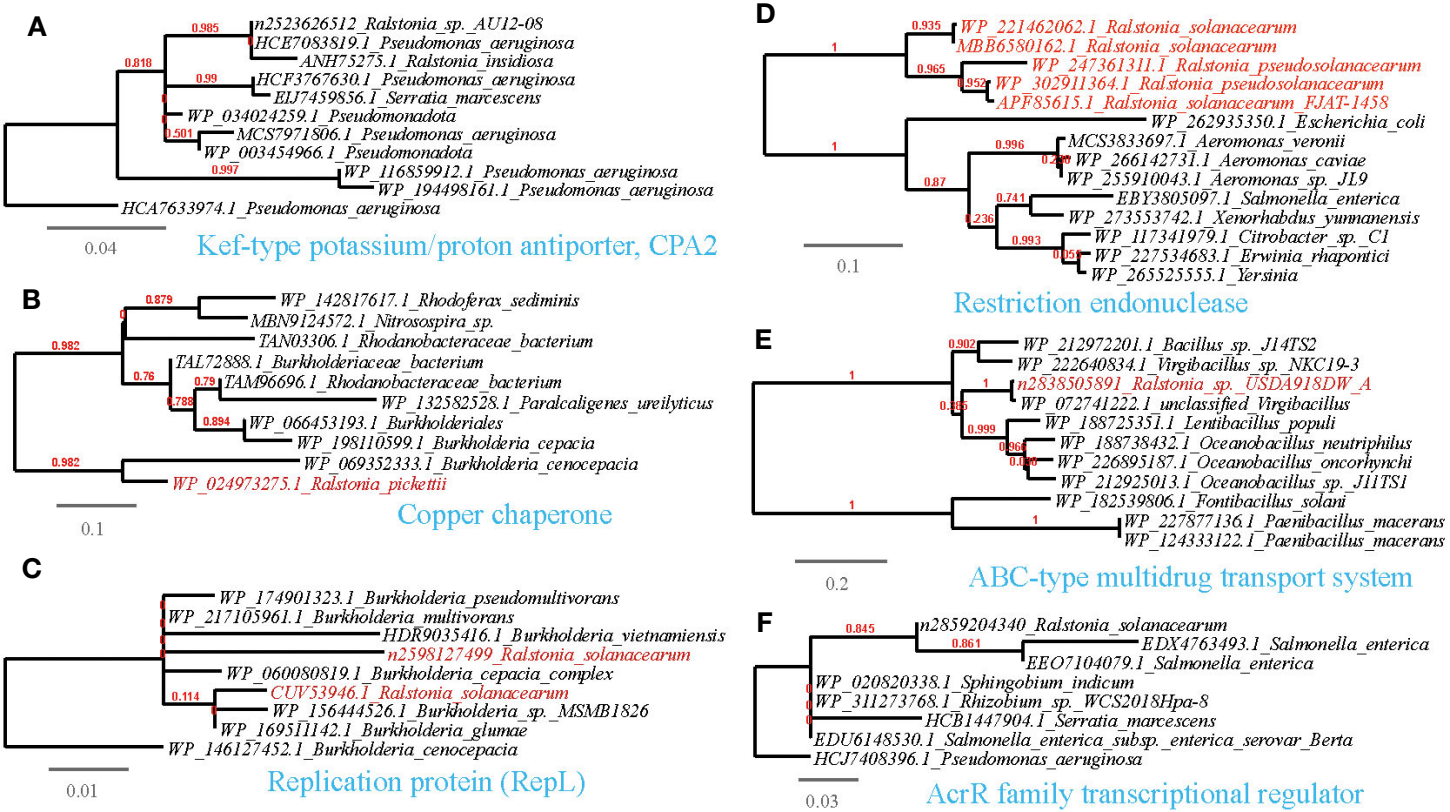
Phylogenetic analyses of the representative HGT genes of *Ralstonia* spp. related with abiotic stress resistance in this study (marked with red color) with the existing sequences from Genbank database (constructed with PhyML, the branch length is proportional to the number of substitutions per site): Kef-type potassium/proton antiporter, CPA2 **(A)**, Copper chaperone **(B)**, Replication protein (RepL) **(C)**, Restriction endonuclease **(D)**, ABC-type multidrug transport system **(E)**, AcrR family transcriptional regulator **(F)**.

Following the results of MENs and the random forest analysis, we sought to explore if the microbial taxa present in the MENs/random forest and correlated with *Ralstonia* spp. had the potential to share functional genes. To accomplish this, we utilized the available genomes of *Ralstonia* spp. isolates to detect putative horizontal gene transfer (HGT) events from the correlating taxa in MENs. Interestingly, our analysis revealed 480 entries of such HGT events (**Supplementary Table S3**). To confirm the accuracy of HGT inference, we performed phylogenetic reconstruction on representative horizontally transferred genes (HTGs) (**Figures 6**–**9**). Among the identified HTGs, several putatively confer adaptive functions to opportunistic plant-associated pathogenic microorganisms, which include:

#### 3.5.1 Attachment and entry into host tissue

Plant-associated pathogens rely on the degradation of plant cell wall structures for energy and to gain entry into the host. This study identified several HGT events related to these functions. Specifically, Type IV secretion system proteins (VirB2, VirB5, VirB10, VirD4), and type III effector AvrPtoB and XopAR were found to be shared between *Ralstonia* spp. and plant-associated bacteria belonging to *Pseudomonas, Xanthomonas* and *Sphingobium* (**Supplementary Table S3**; **Figures 6A, B**). Notably, subtle modifications to these proteins can enable evasion from biological attacks, as seen in *Pseudomonas aeruginosa*, where modifications to the pilus, O-antigen, and type IV pili glycosylation help occlude phage attacks. In our study, we observed shared proteins, such as LPS O-acetylase and glycosyl transferases, between *Ralstonia* and *Pseudomonas*, further supporting their role in evading host defenses (**Supplementary Table S3**; **Figure 6C**).

Apart from the Type IV secretion system, other crucial mechanisms have been identified in plant-associated microbes. For example, *Pseudomonas* spp. utilize their flagellar apparatus to attach to preferred sites on the plant surface and reach more favorable locations (Haefele and Lindow, 1987). Consistent with our findings, we also found shared cell filamentation proteins between *Ralstonia* spp. and *Escherichia coli*, supporting the importance of these proteins in host attachment (**Figure 6D**). Moreover, virulence proteins involved in host attachment and pathogenicity, such as the CheY-like chemotaxis protein, Flp pilus assembly protein TadG, surface-exposed virulence protein and iron-regulated filamentous hemagglutinin (Sun et al., 2016), were shared between *Ralstonia* spp. and plant-associated microbes in the MENs, such as *Pseudomonas* (**Supplementary Table S3**).

In addition to the apparatus implicated in host attachment, we found that esterase/lipase genes, which are involved in breaking down the cuticle layers of the plant cell wall regulated by quorum sensing (QS), were shared between *R.solanacearum* and plant-associated microorganisms such as *Bradyrhizobium* and *Pseudomonas* (**Supplementary Table S3**). These findings support previous research highlighting the role of these genes in enzymatically breaking down the plant cell wall and evading plant defenses (Voigt et al., 2005; Devescovi et al., 2007; Feng et al., 2009; Liu et al., 2018). Another significant finding is the prediction of the transmission of a mannan endo-1,4-beta-mannosidase between *R.solanacearum* and *Sorangium cellulosum* (**Supplementary Table S3**). This mannitol metabolic enzyme is associated with growth and pathogenicity in phytopathogens (Vélëz et al., 2008) and has the ability to trim host glycoproteins (Reichenbach et al., 2018). Moreover, the AttM/AiiB family protein, which possesses acyl-HSL-degrading activity that can regulate the QS system and virulence of pathogens (Huang et al., 2012), is shared between *Ralstonia* and *Bradyrhizobium* (see **Supplementary Table S3**, **Figure 6E**). These findings highlight the potential role of this protein in modulating pathogenicity and intercellular communication.

#### 3.5.2 Ingestion and utilization of nutrients from the host

The availability of nutrient compounds on plant surfaces plays a critical role in the colonization of bacterial populations. Consistent with this, we identified the HGT of a Ca^2+^-binding protein of the RTX (repeats in toxin) family among phytopathogens, including *R.solanacearum* (causing wilt disease), *Pseudomonas syringae* (causing wildfire disease), and *Xanthomonas arboricola* (causing spot disease), on a cross-class level (**Figure 7A**). This protein is known to cause the leakage of host cellular content by forming pores on targeted host membranes (Ostolaza et al., 2019). Furthermore, we discovered the transfer of a Zona occludens toxin (Zot) between *R.solanacearum* and *Ralstonia* phage RSS1 (**Figure 7B**). Zot possesses the ability to reversibly alter the junctions of cell surface, thereby allowing the passage of macromolecules through mucosal barriers (Wang et al., 2011). This finding suggests a potential role for Zot in nutrient acquisition and colonization strategies of *R.solanacearum*. Our findings also align with previous researches that highlight the role of the HGT genes in the enzymatic breakdown of the plant cell wall and to evade plant defenses (Voigt et al., 2005; Devescovi et al., 2007; Feng et al., 2009; Liu et al., 2018).

On the other hand, plant-associated osmotrophic microbes heavily rely on their array of plasma membrane transporters for nutrient acquisition. Through horizontal gene transfer (HGT), these microbes can acquire novel transporter genes, expanding their nutrient repertoire and allowing them to thrive in different ecological niches. This competitive advantage over other microorganisms in the same environment is facilitated by the acquisition of nutrient transporters (Richards and Talbot, 2013). We identified abundant HGT events of transporter-encoding genes acquired by *Ralstonia* from other microorganisms present in the molecular ecological network (**Supplementary Table S3**, **Figures 7C, D**). The HTGs we identified are involved in the transport of various nutrients, including the PST family polysaccharide transporter (*Ralstonia-Escherichia/Pseudomonas*), ABC-type phosphate transporter (*Ralstonia-Pseudomona*), ABC-type sugar transport system ATPase (*R.solanacearum-Xanthomonas arboricola*), NitT/TauT family transport system (*Ralstonia-Pseudomonas*), polyamine/organocation transporter (*Ralstonia-Pseudomonas*), cyanate permease (*Ralstonia-Pseudomonas*), sugar phosphate permease (*Ralstonia*-Bradyrhizobiaceae) and sugar-phosphatase (*Ralstonia-Pseudomonas*) (**Supplementary Table S3**).

#### 3.5.3 Counteract, subvert, or manipulate host pathways

To counteract, subvert, or manipulate host pathways, plants employ various defense responses when they detect invading pathogens. These responses include the production of reactive oxygen species (ROS) and the secretion of antimicrobial toxins. In the successful infection of certain biotrophic and hemibiotrophic phytopathogens, inhibiting the plant’s oxidative burst is crucial. Phytopathogens such as Pezizomycotina produce both intracellular and secreted antioxidants acquired through HGT from nearby microbes (Johnson et al., 2002; Klotz and Loewen, 2003). In the current study, we identified HGT events involving antioxidant enzymes that neutralize ROS (**Supplementary Table 3**; **Figure 8A**). For instance, we found a thioredoxin reductase shared between *Ralstonia* and *Streptomyces*, a thioredoxin, a multimeric flavodoxin WrbA and an alkylhydroperoxidase YurZ shared between *Ralstonia* and *Pseudomonas*, and a shared glutathione S-transferase between *Ralstonia* and *Bradyrhizobium*. Additionally, several mobile genetic elements (MGEs), such as transposase and phage components (see **Supplementary Table 3**, **Figures 8B-F**), were also identified. Besides, TrfA protein essential to plasmid replication (Chou et al., 2014), was observed to be share between *Ralstonia pickettii-Xanthomonas oryzae*. Furthermore, the HGT of a zeta toxin, which stabilizes the resistance plasmid and enhances pathogenicity (Karthika et al., 2021), was observed between *R.solanacearum* and *Xanthomonas citri* pv. malvacearum. *Ralstonia* and associated phage share the reverse transcriptase (RT) and viral RNase H protein. These proteins, along with other viral sequences, can provide host immune defense (Moelling et al., 2017). Additionally, the HGT of peptide deformylase, an essential bacterial metalloenzyme and a promising target for antibiotic action (Zhou et al., 2005), has been observed between *Ralstonia* and *Pseudomonas*. Overall, these findings highlight the importance of counteracting, subverting, or manipulating host pathways for successful pathogen infection, as well as the significant role played by HGT and MGEs in facilitating these processes (**Supplementary Table 3**).

#### 3.5.4 Abiotic stress resistance

Plant-surface microorganisms often face challenging abiotic stresses caused by climate fluctuations, including osmotic stress, sun exposure, and exposure to antimicrobial substances like antibiotics and metal ions. HGT events play a crucial role in enabling these microorganisms to adapt to such harsh conditions.

We have identified several examples of HGT events associated with osmotic stress resistance (**Supplementary Table S3**, **Figure 9A**). These events include the sharing of genes such as the phosphate ABC transporter substrate-binding protein, PhoT family, Na^+^/H^+^-dicarboxylate symporter and Kef-type potassium/proton antiporter, CPA2 family (TC 2.A.37.1), predominantly between *Ralstonia* and *Pseudomonas*. Moreover, we have observed HGT events associated with DNA repair and radiation resistance (**Supplementary Table S3**; **Figures 9B, C**), such as the sharing of endonucleases between *Ralstonia* and plant-associated bacteria such as *Xanthomonas*, *Pseudomonas*, and *Sphingobium*. Additionally, other shared genes have been identified, including UvrD/REP helicase (*Ralstonia-Pseudomonas*), S1/P1 Nuclease (*Ralstonia-Bradyrhizobium*), bacterial DNA primase (*Ralstonia-Ralstonia* virus), alkylation response protein with flavin-dependent DNA repair activity (Bowles et al., 2008), replication protein RepL (*Ralstonia-Escherichia*), and DNA repair protein RadC (*Ralstonia-Pseudomonas*). Furthermore, we have found genes conferring resistance to antimicrobial substances containing metal ions (**Supplementary Table S3**; **Figure 9D**), such as the copper chaperone shared between *Ralstonia* and *Rhodanobacter*. and Mg^2+^-importing ATPase shared between *Ralstonia* and *Pseudomonas*.

Regarding antimicrobial resistance, we have identified HGT events involving various mechanisms related to the inactivation and efflux of antibiotics or toxic organic compounds (**Supplementary Table S3**; **Figure 9E**). These genes include the ABC-type multidrug transport system (*Ralstonia-Streptomyces*), L-carnitine dehydratase (*Ralstonia-Pseudomonas*), camphor resistance protein CrcB (*Ralstonia-Pseudomonas*), aromatic ring-opening dioxygenase LigB (*Ralstonia-Streptomyces*). beta-lactamases (*Ralstonia-Bradyrhizobium/Pseudomonas*). Moreover, we have observed the sharing of the transcriptional anti-activator TraM, which directly regulates quorum sensing in plant pathogens, between *Ralstonia* and *Xanthomonas* (Alakavuklar et al., 2021); Additionally, the transcription regulator AlpA and AcrR that respond to environmental stressors, such as heavy metals (Twibell et al., 2017), is shared between *Ralstonia* and *Klebsiella*. These proteins serve as further examples of HGT events associated with a comprehensive response to abiotic stress resistance (**Supplementary Table S3**; **Figure 9F**).

## 4 Discussion

The objective of this research was to examine the intricate relationships between soil microbial populations and the incidence of *R.solanacearum* invasion in agricultural areas. Our goals were to fill in the gaps in our understanding of the variables affecting pathogenic *R.solanacearum* growth and how microbial communities react to its presence. By accomplishing these goals, we hope to offer a fresh approach and offer theoretical justification for researching antagonistic microbial resources that target *R.solanacearum* and examining microbial resources in crop soil. Our work contributed to the body of knowledge by expanding on earlier studies that looked at the connections between microbial populations, disease incidence, and soil qualities. Nevertheless, little study has been done on the regulation mechanisms of environmental influences, particularly how dietary enhancement and varying nutritional levels affect the suppression of illness (Guo et al., 2022). Furthermore, while research on impact of bacterial wilt disease incidence has been conducted, little focus has been placed on comprehending the complex web of interactions involving other microorganisms in the context of this pathogenic invasion, such as bacteria, fungi, and archaea.

The presence of pathogenic *R.solanacearum* in the soil has a profound impact on the microbial community. Our research highlights the contrasting microbial community composition between affected and healthy plants, as evidenced by reduced network stability. These results consistented with other research showing how phytopathogens can reduce microbial diversity and change the organization of communities (Qu et al., 2020; Tan et al., 2022). The decline in diversity may be attributed to the competitive advantage gained by phytopathogens, potentially through toxin release or resource competition (Bais et al., 2003; Fu et al., 2017). Additionally, changes in soil conditions induced by pathogen intrusion, such as shifts in pH or nutrient availability, can also contribute to alterations in the microbial community structure (Zhang et al., 2016).

In this study, we found that within the same field, healthy and ill plants had rather different microbiome features. Actinobacteria have drawn a lot of attention because of their capacity to inhibit plant pathogens and generated a variety of compounds, including hydrocyanic acid, siderophores, and antifungal compounds (Palaniyandi et al., 2013; Anwar et al., 2016; Sreevidya et al., 2016). They also improved nutrient absorption, which helped to promote plant growth, and generated a great deal of antibiotics; over 45% of all antibiotics on the market were produced by them, and many of these were useful in treating plant diseases (Cheng et al., 2010; Palaniyandi et al., 2011; Liu et al., 2012). In our study, we observed a higher abundance of Actinobacteria in the CK group, suggesting that diseased plants may recruit Actinobacteria to resist pathogens, thereby increasing their abundance in the soil associated with the prevention of disease invasion. A similar effect may explain the enrichment of antagonistic Firmicutes taxa in CK3 (Ahmed et al., 2022a; Jaffar et al., 2023). Ammonia-oxidizing archaea, such as Nitrososphaeraceae, and methanotrophic archaea, such as *Methanococcus*, were more enriched in the CK groups. These microorganisms play a role in the oxidation of ammonia to nitrite and can also reduce ammonia volatilization, thereby increasing the utilization of NH_4_^+^-N by plants (Holmes et al., 1995; Wang et al., 2018).

*Burkholderia*’s ability to promote plant development, perform as an effective biological control agent, and have antimicrobial qualities have all been the subject of much research. (Kunakom and Eustaquio, 2019). It has been found to act as an antagonist against black spot disease in cherries and secrete beneficial compounds that protect bananas from wilt disease (Xu et al., 2020; Ding et al., 2021). Consistent with previous research, our study observed a higher abundance of *Burkholderia* in the microbial MEN of healthy samples compared to diseased ones, suggesting that *Burkholderia* is a beneficial bacterium for tobacco. Therefore, it can be inferred that Burkholderia has the potential to protect crops from plant diseases.

This study examines the effects of pathogenic *R.solanacearum* on soil microbial communities and highlights various aspects such as changes in network scale, keystone species dynamics, community interactions, and stability. Understanding these dynamics is essential for mitigating wilt disease outbreaks and maintaining the health of microbial ecosystems. According to earlier studies, community stability and persistence were greatly influenced by their interactions and connections, with network structure being essential to community resilience to invasion (Xu et al., 2020; Ji et al., 2021). Improved community stability and anti-interference capabilities were facilitated by complex microbial networks in the soil (Yang et al., 2019; Xu et al., 2020). Furthermore, increased connectivity within a network fostered higher collaboration and functional redundancy among healthy plant soil communities (Mougi and Kondoh, 2012; Zhang et al., 2022). These intricate networks had the ability to successfully adjust changes in the environment and prevented soil-transmitted diseases from infecting plants (Yang et al., 2017; Tao et al., 2018). Highly diverse microbial communities often exhibit enhanced resistance to pathogen infection, making the soil itself more resilient (Mallon et al., 2015). However, in the case of diseased wheat, the disruption of the mutualistic relationship among fungal communities leads to reduced complexity and stability within the community, creating a more favorable environment for pathogen infection (Nwokolo et al., 2021).

The impact of pathogenic *R.solanacearum* on soil microbial communities is examined in this study, with a focus on its transformative influence. The infection leads to a reduction in the complexity of microbial communities, as indicated by a decrease in network scale (**Figure 2**), which has been observed in previous studies as well (Urich et al., 2008; Banerjee et al., 2018). Keystone species play crucial roles in sustaining ecosystem function and resilience (Banerjee et al., 2018). Conversely, the increase in keystone species within community networks may be a response to the invasion of *R.solanacearum*, potentially suppressing its growth or facilitating the establishment of other beneficial microbial taxa (Tan et al., 2022). Noteworthy examples include Haloarculaceae, Halobacteriaceae, and Halorubraceae, which degrade cellulose, lignin, chitin, and biopolymers (Moopantakath et al., 2023), and could contribute significantly to the suppressing of the wilt disease outbreaks. Moreover, cohesion analysis reveals a shift in microbial interactions, with intensified competition in healthy soils and increased cooperation in infected soils (**Figure 3A**). This shift implies changes in microbial community composition and structure (Chapelle et al., 2016). The analysis also suggests that prokaryotic communities in healthy soils exhibit greater stability compared to infected soils (**Figure 3B**), potentially indicating the detrimental impact of *R.solanacearum* on the stability of these microbial communities (Yan and Jr. Nelson, 2020; Saengchan et al., 2022).

The interactions among bacteria, fungi, and archaea in soil ecosystems play a crucial role in microbial communication networks, which are essential for the balance and functioning of these habitats (Deng et al., 2012). Our study specifically focuses on the significant role of inter-domain interactions in soil ecosystem dynamics. The intrusion of pathogens, such as *R.solanacearum*, disrupts these interactions and has transformative effects on the microbial community structure. Using the MEN approach, we investigated complex bacterial-fungal associations in healthy soils. However, upon infection, we observed an increase in the connections between *R.solanacearum* and prokaryotic species. This phenomenon suggests that pathogenic fungi may stimulate the growth of specific bacterial species, potentially promoting or suppressing fungal growth. This insight provides valuable information for regulating the expansion and virulence of *R.solanacearum* (Tan et al., 2021). Consistent with our findings, previous research has highlighted the crucial role of prokaryotes in soil health, particularly in controlling phytopathogens (Morse et al., 2003; Niu et al., 2017). Furthermore, Deng et al. (Deng et al., 2022) showed in a glasshouse experiment that community composition changes, not just the quantity of added biocontrol strains, were the main cause of the suppressiveness of rhizosphere bacterial communities. This finding underscores the importance of understanding the intricate dynamics and interactions within microbial communities for effective disease management strategies.

The current study has also revealed the presence of abundant horizontally transferred genes (HTGs) in opportunistic plant-associated pathogenic microorganisms, indicating their role in conferring adaptive functions. This highlights the significance of horizontal gene transfer (HGT) in shaping the genetic repertoire and adaptive traits of plant-associated bacteria, including *Ralstonia* spp., particularly in relation to host colonization and pathogenicity. The shared virulence proteins highlight their potential role in intercellular communication and host manipulation (**Supplementary Table S3**; **Figure 6C**). Specifically, VirB2 and VirB5 form pilus structures extending from the extracellular surface, while VirB10 forms the outer membrane channel inserting in both the inner and outer membranes (Mossey et al., 2010). VirD4 serves as an associated substrate recognition receptor (Wallden et al., 2010). Furthermore, the Type III effector AvrPtoB with E3 ubiquitin ligase activity and type III effector candidate XopAR can suppress plant cell death and immunity (Abramovitch et al., 2006).

Our study further reinforces the significance of HGT in shaping nutrient acquisition strategies in plant-associated microorganisms. These findings highlight the importance of HGT in shaping the evolution of microbial nutrient acquisition strategies. They also suggest that plant-associated microbes may share common mechanisms for acquiring nutrients from their environments (Bachhawat et al., 2013). By acquiring transporter genes through HGT, plant-associated microbes can enhance their nutrient uptake capabilities, enabling them to adapt to diverse nutritional conditions. This adaptability is central to their ability to establish symbiotic relationships with plants and exploit their shared environments, ultimately leading to their ecological success. Collectively, these findings underscore the common features and mechanisms utilized by plant-associated microorganisms for interacting with their host plants. The identification of shared proteins involved in vital processes such as cellular attachment, virulence, and enzymatic breakdown of the plant cell wall indicates the presence of conserved strategies and adaptations in the microbial ecology of plant-associated pathogens.

## 5 Conclusions

This study aims to bridge the knowledge gaps by investigating the complex interactions between soil microbial communities and *R.solanacearum* infection in crop fields. We aim to propose a novel strategy for studying microbial resources in crop soil and exploring antagonistic microbial resources targeting *R.solanacearum*. The significant differences in microbiome characteristics between healthy and diseased plants underscore the impact of *R.solanacearum* on the microbial community, leading to reduced diversity and altered community structure. These changes may be attributed to competitive advantage gained by the pathogen and shifts in soil conditions induced by the pathogen intrusion. Our findings emphasize the significant influence of phytopathogen infestations on soil microbial communities, leading to reduced diversity and richness. These outcomes have important implications for plant health and agricultural productivity, given the crucial role of soil microbial communities in nutrient cycling, soil structure, and plant-microbe interactions. Bacteria and archaea were more vulnerable than fungi when the bacterial wilt outbreak with the reduce of soil phosphorus. Network analysis indicated that the interactions among pathogenic *R.solanacearm* and other microbial members were more in the networks of diseased than healthy soils. The presence of certain bacteria (Comamonadaceae) and archaea (Halorubrum, Halobacteriaceae, Haloarculaceae) enriched in healthy plant rhizosphere soils, could potentially reduce the occurrence of pathogenic *R.solanacearm*. Conversely, certain archaea (Natrialbaceae) were most abundant in diseased samples, which could contribute to the outbreaks of bacterial wilt. To understand the fundamental processes causing these impacts and to create management plans for soil-borne plant diseases, more research is required.

## Data availability statement

The data presented in this study are deposited in the National Genomics Data Center (cncb.ac.cn) under Bioproject repository, accession number PRJCA020669. Other data are available upon request from the corresponding author(s).

## Author contributions

YX: Writing – original draft, Data curation. SZ: Data curation, Investigation, Writing – original draft, Conceptualization. HL: Formal analysis, Methodology, Writing – original draft. KT: Data curation, Writing – original draft. SW: Data curation, Writing – original draft. YL: Formal analysis, Writing – original draft. FY: Formal analysis, Writing – original draft. ZH: Formal analysis, Writing – original draft. LJL: Formal analysis, Writing – original draft. LZL: Visualization, Writing – review & editing. DM: Conceptualization, Visualization, Writing – review & editing. HY: Supervision, Writing – review & editing. YW: Writing – review & editing, Visualization.

## References

[B1] (1978). Soil physical and chemical analysis. Institute Soil Sci. Chin. Acad. Sci.

[B2] Abramovitch R. B. Janjusevic R. Stebbins C. E. Martin G. B. (2006). Type iii effector avrptob requires intrinsic e3 ubiquitin ligase activity to suppress plant cell death and immunity. Proc. Natl. Acad. Sci. U. S. A. 103, 2851–2856. doi: 10.1073/pnas.0507892103 16477026 PMC1413779

[B3] Addy H. S. Askora A. Kawasaki T. Fujie M. Yamada T. (2012). Utilization of filamentous phage φrsm3 to control bacterial wilt caused by *ralstonia solanacearum* . Plant Dis. 96, 1204–1209. doi: 10.1094/PDIS-12-11-1023-RE 30727062

[B4] Ahmed W. Dai Z. Zhang J. Li S. Ahmed A. Munir S. Liu Q. (2022a). Plant-microbe interaction: mining the impact of native bacillus amyloliquefaciens ws-10 on tobacco bacterial wilt disease and rhizosphere microbial communities. Microbiol. Spectr. 10, e147122. doi: 10.1128/spectrum.01471-22 PMC9430121 35913211

[B5] Ahmed W. Yang J. Tan Y. Munir S. Liu Q. Zhang J.. (2022b). *ralstonia solanacearum*, a deadly pathogen: revisiting the bacterial wilt biocontrol practices in tobacco and other solanaceae. Rhizosphere 21, 100479. doi: 10.1016/j.rhisph.2022.100479

[B6] Alakavuklar M. A. Heckel B. C. Stoner A. M. Stembel J. A. Fuqua C. (2021). Motility control through an anti-activation mechanism in agrobacterium tumefaciens. Mol. Microbiol. 116, 1281–1297. doi: 10.1111/mmi.14823 34581467 PMC8690355

[B7] Anwar S. Ali B. Sajid I. (2016). Screening of rhizospheric actinomycetes for various in-vitro and in-vivo plant growth promoting (pgp) traits and for agroactive compounds. Front. Microbiol. 7. doi: 10.3389/fmicb.2016.01334 PMC5002418 27621724

[B8] Bachhawat A. K. Thakur A. Kaur J. Zulkifli M. (2013). Glutathione transporters. Biochim. Et Biophys. Acta (Bba) Gen. Subj. 1830, 3154–3164. doi: 10.1016/j.bbagen.2012.11.018 23206830

[B9] Bader G. D. Hogue C. W. (2003). An automated method for finding molecular complexes in large protein interaction networks. BMC Bioinf. 4. doi: 10.1186/1471-2105-4-2 PMC149346 12525261

[B10] Bais H. P. Vepachedu R Gilroy S. Callaway R. M. Vivanco J. M. (2003). Allelopathy and exotic plant invasion: from molecules and genes to species interactions. Science 301 (5638), 1377–1380. doi: 10.1126/science.1083245 12958360

[B11] Bakker P. A. H. M. Pieterse C. M. J. de Jonge R. Berendsen R. L. (2018). The soil-borne legacy. Cell 172, 1178–1180. doi: 10.1016/j.cell.2018.02.024 29522740

[B12] Bankevich A. Nurk S. Antipov D. Gurevich A. A. Dvorkin M. Kulikov A. S.. (2012). Spades: a new genome assembly algorithm and its applications to single-cell sequencing. J. Comput. Biol. 19, 455–477. doi: 10.1089/cmb.2012.0021 22506599 PMC3342519

[B13] Banerjee S. Schlaeppi K. van der Heijden M. G. A. (2018). Keystone taxa as drivers of microbiome structure and functioning. Nat. Rev. Microbiol. 16, 567–576. doi: 10.1038/s41579-018-0024-1 29789680

[B14] Bonanomi G. Lorito M. Vinale F. Woo S. L. (2018). Organic amendments, beneficial microbes, and soil microbiota: toward a unified framework for disease suppression. Annu Rev Phytopathol. 56, 1–20. doi: 10.1146/annurev-phyto-080615-100046 29768137

[B15] Bowles T. Metz A. H. O'Quin J. Wawrzak Z. Eichman B. F. (2008). Structure and dna binding of alkylation response protein aidb. Proc. Natl. Acad. Sci. U. S. A. 105, 15299–15304. doi: 10.1073/pnas.0806521105 18829440 PMC2563087

[B16] Chapelle E. Mendes R. Bakker P. A. H. M. Raaijmakers J. M. (2016). Fungal invasion of the rhizosphere microbiome. Isme J. 10, 265–268. doi: 10.1038/ismej.2015.82 26023875 PMC4681858

[B17] Cheng J. Yang S. H. Palaniyandi S. A. Han J. S. Yoon T.. (2010). Azalomycin f complex is an antifungal substance produced by *streptomyces malaysiensis* mjm1968 isolated from agricultural soil. J. Korean Soc. Appl. Biol. Chem. 53, 545–552. doi: 10.3839/jksabc.2010.084

[B18] Chou H. H. Delaney N. F. Draghi J. A. Marx C. J. (2014). Mapping the fitness landscape of gene expression uncovers the cause of antagonism and sign epistasis between adaptive mutations. PloS Genet. 10, e1004149. doi: 10.1371/journal.pgen.1004149 24586190 PMC3937219

[B19] Dai Z. L. Ahmed W. Yang J. Yao X. Y. Zhang J. H. Wei L. F.. (2023). Seed coat treatment by plant-growth-promoting rhizobacteria 13-6 enhances maize yield and changes rhizosphere bacterial communities. Biol. Fertility Soils 59 (3), 317–331. doi: 10.1007/s00374-023-01703-x

[B20] Deng Y. Jiang Y. Yang Y. He Z. Luo F. Zhou J. Z. (2012). Molecular ecological network analyses. BMC Bioinf. 13, 113. doi: 10.1186/1471-2105-13-113 PMC3428680 22646978

[B21] Deng X. Zhang N. Li Y. Zhu C. Qu B. Liu H.. (2022). Bio-organic soil amendment promotes the suppression of ralstonia solanacearum by inducing changes in the functionality and composition of rhizosphere bacterial communities. New Phytol. 235, 1558–1574. doi: 10.1111/nph.18221 35569105

[B22] Devescovi G. Bigirimana J. Degrassi G. Cabrio L. LiPuma J. J. Kim J.. (2007). Involvement of a quorum-sensing-regulated lipase secreted by a clinical isolate of burkholderia glumae in severe disease symptoms in rice. Appl. Environ. Microbiol. 73, 4950–4958. doi: 10.1128/AEM.00105-07 17557855 PMC1951028

[B23] Ding L. Xu L. Chu X. Yang L. Zhu H. Huang J. (2021). Dissimilarity analysis of microbial communities in the rhizosphere and tissues of diseased and healthy cherry trees (cerasus pseudocerasus). Can. J. Plant Pathol. 43, 612–621. doi: 10.1080/07060661.2020.1861101

[B24] Dixon P. (2003). Vegan, a package of r functions for community ecology. J. Veg. Sci. 14, 927–930. doi: 10.1658/1100-9233(2003)014[0927:VAPORF]2.0.CO;2

[B25] Edgar R. C. (2004). MUSCLE: multiple sequence alignment with high accuracy and high throughput. Nucleic Acids Res. 32 (5), 1792–1797. doi: 10.1093/nar/gkh340 15034147 PMC390337

[B26] Edwards J. Johnson C. Santos-Medellin C. Lurie E. Podishetty N. K. Bhatnagar S.. (2015). Structure, variation, and assembly of the root-associated microbiomes of rice. Proc. Natl. Acad. Sci. U. States America 112 (8), E911–E920. doi: 10.1073/pnas.1414592112 PMC4345613 25605935

[B27] Faust K. Raes J. (2016). Conet app: inference of biological association networks using cytoscape. F1000Research 5, 1519. 27853510 10.12688/f1000research.9050.1 PMC5089131

[B28] Feng J. Wang F. Liu G. Greenshields D. Shen W. Kaminskyj S.. (2009). Analysis of a blumeria graminis-secreted lipase reveals the importance of host epicuticular wax components for fungal adhesion and development. Mol. Plant-Microbe Interact. 22, 1601–1610. doi: 10.1094/MPMI-22-12-1601 19888825

[B29] Fu L. Penton C. R. Ruan Y. Shen Z. Xue C. Li R.. (2017). Inducing the rhizosphere microbiome by biofertilizer application to suppress banana fusarium wilt disease. Soil Biol. Biochem. 104, 39–48. doi: 10.1016/j.soilbio.2016.10.008

[B30] Guindon S. Dufayard J. F. Lefort V. Anisimova M. Hordijk W. Gascuel O. (2010). New algorithms and methods to estimate maximum-likelihood phylogenies: assessing the performance of PhyML 3.0. Syst. Biol. 59 (3), 307–321. doi: 10.1093/sysbio/syq010 20525638

[B31] Guo S. Tao C. Jousset A. Xiong W. Wang Z. Shen Z.. (2022). Trophic interactions between predatory protists and pathogen-suppressive bacteria impact plant health. Isme J. 16, 1932–1943. doi: 10.1038/s41396-022-01244-5 35461357 PMC9296445

[B32] Haefele D. M. Lindow S. E. (1987). Flagellar motility confers epiphytic fitness advantages upon pseudomonas syringae. Appl. Environ. Microbiol. 53, 2528–2533. doi: 10.1128/aem.53.10.2528-2533.1987 16347469 PMC204140

[B33] Holmes A. J. Costello A. Lidstrom M. E. Murrell J. C. (1995). Evidence that particulate methane monooxygenase and ammonia monooxygenase may be evolutionarily related. FEMS Microbiol. Lett. 132, 203–208. doi: 10.1016/0378-1097(95)00311-R 7590173

[B34] Huang W. Lin Y. Yi S. Liu P. Shen J. Shao Z.. (2012). Qsdh, a novel ahl lactonase in the rnd-type inner membrane of marine pseudoalteromonas byunsanensis strain 1a01261. PloS One 7, e46587. doi: 10.1371/journal.pone.0046587 23056356 PMC3466314

[B35] Jacomy M. Venturini T. Heymann S. Bastian M. (2014). Forceatlas2, a continuous graph layout algorithm for handy network visualization designed for the gephi software. PloS One 9. doi: 10.1371/journal.pone.0098679 PMC4051631 24914678

[B36] Jaffar N. S. Jawan R. Chong K. P. (2023). The potential of lactic acid bacteria in mediating the control of plant diseases and plant growth stimulation in crop production - a mini review. Front. Plant Sci. 13. doi: 10.3389/fpls.2022.1047945 PMC9880282 36714743

[B37] Ji L. Tian L. Nasir F. Chang J. Chang C. Zhang J.. (2021). Impacts of replanting american ginseng on fungal assembly and abundance in response to disease outbreaks. Arch. Microbiol. 203, 2157–2170. doi: 10.1007/s00203-021-02196-8 33616683 PMC8205870

[B38] Jiang G. Wei Z. Xu J. Chen H. Zhang Y. She X.. (2017). Bacterial wilt in China: history, current status, and future perspectives. Front. Plant Sci. 8. doi: 10.3389/fpls.2017.01549 PMC5601990 28955350

[B39] Johnson C. H. Klotz M. G. York J. L. Kruft V. McEwen J. E. (2002). Redundancy, phylogeny and differential expression of histoplasma capsulatum catalases. Microbiology-(Uk) 148, 1129–1142. doi: 10.1099/00221287-148-4-1129 11932457

[B40] Karthika A. Ramachandran B. Chitra J. Prabhu D. Rajamanikandan S. Veerapandiyan M.. (2021). Molecular dynamics simulation of toxin-antitoxin (ta) system in acinetobacter baumannii to explore the novel mechanism for inhibition of cell wall biosynthesis: zeta toxin as an effective therapeutic target. J. Cell. Biochem. 122, 1832–1847. doi: 10.1002/jcb.30137 34448250

[B41] Kechin A. Boyarskikh U. Kel A. Filipenko M. (2017). Cutprimers: a new tool for accurate cutting of primers from reads of targeted next generation sequencing. J. Comput. Biol. 24, 1138–1143. doi: 10.1089/cmb.2017.0096 28715235

[B42] Klotz M. G. Loewen P. C. (2003). The molecular evolution of catalatic hydroperoxidases: evidence for multiple lateral transfer of genes between prokaryota and from bacteria into eukaryota. Mol. Biol. Evol. 20, 1098–1112. doi: 10.1093/molbev/msg129 12777528

[B43] Kunakom S. Eustaquio A. S. (2019). *burkholderia* as a source of natural products. J. Nat. Prod. 82, 2018–2037. doi: 10.1021/acs.jnatprod.8b01068 31294966 PMC6871192

[B44] Langmead B. Salzberg S. L. (2012). Fast gapped-read alignment with bowtie 2. Nat. Methods 9, 354–357. doi: 10.1038/NMETH.1923 PMC3322381 22388286

[B45] Letunic I. Bork P. (2021). Interactive Tree Of Life (iTOL) v5: an online tool for phylogenetic tree display and annotation. Nucleic Acids Res. 49 (W1), W293–W296. doi: 10.1093/nar/gkab301 33885785 PMC8265157

[B46] Li C. Ahmed W. Li D. Yu L. Xu L. Xu T.. (2022). Biochar suppresses bacterial wilt disease of flue-cured tobacco by improving soil health and functional diversity of rhizosphere microorganisms. Appl. Soil Ecol. 171, 104314. doi: 10.1016/j.apsoil.2021.104314

[B47] Li W. Godzik A. (2006). Cd-hit: a fast program for clustering and comparing large sets of protein or nucleotide sequences. Bioinformatics 22, 1658–1659. doi: 10.1093/bioinformatics/btl158 16731699

[B48] Liu X. Bolla K. Ashforth E. J. Zhuo Y. Gao H. Huang P.. (2012). Systematics-guided bioprospecting for bioactive microbial natural products. Antonie Van Leeuwenhoek 101, 55–66. doi: 10.1007/s10482-011-9671-1 22086462

[B49] Liu Z. Beskrovnaya P. Melnyk R. A. Hossain S. S. Khorasani S. O'Sullivan L. R.. (2018). A genome-wide screen identifies genes in rhizosphere-associated pseudomonas required to evade plant defenses. Mbio 9 (6), e00433-18. doi: 10.1128/mBio.00433-18 30401768 PMC6222131

[B50] Lu J. Breitwieser F. P. Thielen P. Salzberg S. L. (2017). Bracken: estimating species abundance in metagenomics data. Peerj Comput. Sci. doi: 10.7717/peerj-cs.104 PMC12016282 40271438

[B51] Mallon C. A. van Elsas J. D. Salles J. F. (2015). Microbial invasions: the process, patterns, and mechanisms. Trends Microbiol. 23, 719–729. doi: 10.1016/j.tim.2015.07.013 26439296

[B52] Markowitz V. M. Chen I. A. Palaniappan K. Chu K. Szeto E. Grechkin Y.. (2010). The integrated microbial genomes system: an expanding comparative analysis resource. Nucleic Acids Res. 38, D382–D390. doi: 10.1093/nar/gkp887 19864254 PMC2808961

[B53] Meline V. Caldwell D. L. Kim B. Khangura R. S. Baireddy S. Yang C.. (2023). Image-based assessment of plant disease progression identifies new genetic loci for resistance to *ralstonia solanacearum* in tomato. Plant J. 113, 887–903. doi: 10.1111/tpj.16101 36628472

[B54] Moelling K. Broecker F. Russo G. Sunagawa S. (2017). Rnase h as gene modifier, driver of evolution and antiviral defense. Front. Microbiol. 8. doi: 10.3389/fmicb.2017.01745 PMC5603734 28959243

[B55] Moopantakath J. Imchen M. Anju V. T. Busi S. Dyavaiah M. Martínez-Espinosa R. M.. (2023). Bioactive molecules from haloarchaea: scope and prospects for industrial and therapeutic applications. Front. Microbiol. 14. doi: 10.3389/fmicb.2023.1113540 PMC10102575 37065149

[B56] Mougi A. Kondoh M. (2012). Diversity of interaction types and ecological community stability. Science 337, 349–351. doi: 10.1126/science.1220529 22822151

[B57] Morse H. C. I. McCarty T. Qi C. Torrey T. A. Naghashfar Z. Chattopadhyay SK. (2003). B lymphoid neoplasms of mice: characteristics of naturally occurring and engineered diseases and relationships to human disorders. Adv Immunol. 81, 97–121. doi: 10.1016/s0065-2776(03)81003-9 14711054

[B58] Mossey P. Hudacek A. Das A. (2010). Agrobacterium tumefaciens type iv secretion protein virb3 is an inner membrane protein and requires virb4, virb7, and virb8 for stabilization. J. Bacteriol. 192, 2830–2838. doi: 10.1128/jb.01331-09 20348257 PMC2876495

[B59] Murtagh F. Legendre P. (2014). Ward's hierarchical agglomerative clustering method: which algorithms implement ward's criterion? J. Classif. 31, 274–295. doi: 10.1007/s00357-014-9161-z

[B60] Niu B. Paulson J. N. Zheng X. Kolter R. (2017). Simplified and representative bacterial community of maize roots. Proc. Natl. Acad. Sci. U. S. A. 114, E2450–E2459. doi: 10.1073/pnas.1616148114 28275097 PMC5373366

[B61] Niu B. Wang W. Yuan Z. Sederoff R. R. Sederoff H. Chiang V.L.. (2020). Microbial interactions within multiple-strain biological control agents impact soil-borne plant disease. Front. Microbiol. 11. doi: 10.3389/fmicb.2020.585404 PMC7581727 33162962

[B62] Nwokolo N. L. Enebe M. C. Chigor C. B. Chigor V. N. Dada O. A. (2021). The contributions of biotic lines of defence to improving plant disease suppression in soils: a review. Rhizosphere 19, 100372. doi: 10.1016/j.rhisph.2021.100372

[B63] Ostolaza H. González-Bullón D. Uribe K. B. Martín C. Amuategi J. Fernandez-Martínez X.. (2019). Membrane permeabilization by pore-forming rtx toxins: what kind of lesions do these toxins form? Toxins 11 (6), 354. doi: 10.3390/toxins11060354 31216745 PMC6628442

[B64] Palaniyandi S. A. Yang S. H. Cheng J. H. Meng L. Suh J. W. (2011). Biological control of anthracnose (*colletotrichum gloeosporioides*) in yam by *streptomyces* sp.mjm5763. J. Appl. Microbiol. 111, 443–455. doi: 10.1111/j.1365-2672.2011.05048.x 21714834

[B65] Palaniyandi S. A. Yang S. H. Zhang L. Suh J. (2013). Effects of actinobacteria on plant disease suppression and growth promotion. Appl. Microbiol. Biotechnol. 97, 9621–9636. doi: 10.1007/s00253-013-5206-1 24092003

[B66] Peeters N. Guidot A. Vailleau F. Valls M. (2013). Ralstonia solanacearum , a widespread bacterial plant pathogen in the post-genomic era. Mol. Plant Pathol. 14, 651–662. doi: 10.1111/mpp.12038 23718203 PMC6638647

[B67] Pei W. Nan S. Na H. (2022). Risk area division of meteorological disasters for tobacco farming in chenzhou. J. Xiangnan University Chin. 43, 30–36.

[B68] Qu Q. Zhang Z. Peijnenburg W. J. G. M. Liu W. Lu T. Hu B.. (2020). Rhizosphere microbiome assembly and its impact on plant growth. J. Agric. Food Chem. 68, 5024–5038. doi: 10.1021/acs.jafc.0c00073 32255613

[B69] Rahman S. F. S. A. Singh E. Pieterse C. M. J. Schenk P. M. (2018). Emerging microbial biocontrol strategies for plant pathogens. Plant Sci. 267, 102–111. doi: 10.1016/j.plantsci.2017.11.012 29362088

[B70] Reichenbach T. Kalyani D. Gandini R. Svartström O. Aspeborg H. Divne C.. (2018). Structural and biochemical characterization of the cutibacterium acnes exo-β-1,4-mannosidase that targets the n-glycan core of host glycoproteins. PloS One 13, e204703. doi: 10.1371/journal.pone.0204703 PMC6160142 30261037

[B71] Richards T. A. Talbot N. J. (2013). Horizontal gene transfer in osmotrophs: playing with public goods. Nat. Rev. Microbiol. 11, 720–727. doi: 10.1038/nrmicro3108 24018383

[B72] Saengchan C. Phansak P. Thumanu K. Siriwong S. Le Thanh T. Sangpueak R.. (2022). Resistance induction by salicylic acid formulation in cassava plant against fusarium solani. Plant Pathol. J. 38, 212–219. doi: 10.5423/PPJ.OA.02.2022.0019 35678054 PMC9343910

[B73] Segata N. Izard J. Waldron L. Gevers D. Miropolsky L. Garrett W. S.. (2011). Metagenomic biomarker discovery and explanation. Genome Biol. 12. doi: 10.1186/gb-2011-12-6-r60 PMC3218848 21702898

[B74] Shafi J. Tian H. Ji M. (2017). *bacillus* species as versatile weapons for plant pathogens: a review. Biotechnol. Biotechnol. Equip. 31, 446–459. doi: 10.1080/13102818.2017.1286950

[B75] Sreevidya M. Gopalakrishnan S. Kudapa H. Varshney R. K. (2016). Exploring plant growth-promotion actinomycetes from vermicompost and rhizosphere soil for yield enhancement in chickpea. Braz. J. Microbiol. 47, 85–95. doi: 10.1016/j.bjm.2015.11.030 26887230 PMC4822753

[B76] Sun Y. Y. Chi H. Sun L. (2016). Pseudomonas fluorescens filamentous hemagglutinin, an iron-regulated protein, is an important virulence factor that modulates bacterial pathogenicity. Front. Microbiol. 7. doi: 10.3389/fmicb.2016.01320 PMC4993755 27602029

[B77] Talavera G. Castresana J. (2007). Improvement of phylogenies after removing divergent and ambiguously aligned blocks from protein sequence alignments. Syst. Biol. 56 (4), 564–577. doi: 10.1080/10635150701472164 17654362

[B78] Tan L. Xiao Y. Zeng W. Gu S. Zhai Z. Wu S.. (2022). Network analysis reveals the root endophytic fungi associated with *fusarium* root rot invasion. Appl. Soil Ecol. 178, 104567. doi: 10.1016/j.apsoil.2022.104567

[B79] Tan L. Zeng W. Xiao Y. Li P. Gu S. Wu S.. (2021). Fungi-bacteria associations in wilt diseased rhizosphere and endosphere by interdomain ecological network analysis. Front. Microbiol. 12. doi: 10.3389/fmicb.2021.722626 PMC8450586 34552573

[B80] Tao J. Meng D. Qin C. Liu X. Liang Y. Xiao Y.. (2018). Integrated network analysis reveals the importance of microbial interactions for maize growth. Appl. Microbiol. Biotechnol. 102, 3805–3818. doi: 10.1007/s00253-018-8837-4 29532103

[B81] Twibell B. Somerville K. Manani G. Duszynski M. Wanekaya A. Schweiger P.. (2017). Influence of cntrene(®) c100lm carbon nanotube material on the growth and regulation of escherichia coli. Peerj 5, e3721. doi: 10.7717/peerj.3721 28828284 PMC5564384

[B82] Urich T. Lanzen A. Qi J. Huson D. H. Schleper C. Schuster S. C.. (2008). Simultaneous assessment of soil microbial community structure and function through analysis of the meta-transcriptome. PloS One 3 (6), e2527. doi: 10.1371/journal.pone.0002527 18575584 PMC2424134

[B83] Vélëz H. Glassbrook N. J. Daub M. E. (2008). Mannitol biosynthesis is required for plant pathogenicity by alternaria alternata. FEMS Microbiol. Lett. 285, 122–129. doi: 10.1111/j.1574-6968.2008.01224.x 18549402

[B84] Voigt C. A. Schäfer W. Salomon S. (2005). A secreted lipase of fusarium graminearum is a virulence factor required for infection of cereals. Plant J. For Cell Mol. Biol. 42, 364–375. doi: 10.1111/j.1365-313x.2005.02377.x 15842622

[B85] Wallden K. Rivera-Calzada A. Waksman G. (2010). Type iv secretion systems: versatility and diversity in function. Cell Microbiol. 12, 1203–1212. doi: 10.1111/j.1462-5822.2010.01499.x 20642798 PMC3070162

[B86] Wang H. Li Z. Yumul R. Lara S. Hemminki A. Fender P.. (2011). Multimerization of adenovirus serotype 3 fiber knob domains is required for efficient binding of virus to desmoglein 2 and subsequent opening of epithelial junctions. J. Virol. 85, 6390–6402. doi: 10.1128/JVI.00514-11 21525338 PMC3112237

[B87] Wang X. Xu S. Wu S. Feng S. Bai Z. Zhuang G.. (2018). Effect of *trichoderma viride* biofertilizer on ammonia volatilization from an alkaline soil in northern China. J. Environ. Sci. 66, 199–207. doi: 10.1016/j.jes.2017.05.016 29628087

[B88] Wang R. Zhang H. Sun L. Qi G. Chen S. Zhao X.. (2017). Microbial community composition is related to soil biological and chemical properties and bacterial wilt outbreak. Sci. Rep. 7 (1), 343. doi: 10.1038/s41598-017-00472-6 28336973 PMC5428506

[B89] Wei C. Liu J. Maina A. N. Mwaura F. B. Yu J. Yan C.. (2017). Developing a bacteriophage cocktail for biocontrol of potato bacterial wilt. Virol. Sin. 32, 476–484. doi: 10.1007/s12250-017-3987-6 29168148 PMC6598922

[B90] Wood D. E. Lu J. Langmead B. (2019). Improved metagenomic analysis with kraken 2. Genome Biol. doi: 10.1186/s13059-019-1891-0 PMC6883579 31779668

[B91] Wu D. Wang W. Yao Y. Li H. Wang Q. Niu B.. (2023). Microbial interactions within beneficial consortia promote soil health. Sci. Total Environ. 900, 165801. doi: 10.1016/j.scitotenv.2023.165801 37499809

[B92] Xu Z. Wang M. Du J. Huang T. Liu J. Dong T.. (2020). Isolation of burkholderia sp. Hqb-1, a promising biocontrol bacteria to protect banana against fusarium wilt through phenazine-1-carboxylic acid secretion. Front. Microbiol. 11, 605152. doi: 10.3389/fmicb.2020.605152 33362750 PMC7758292

[B93] Yan H. Jr. Nelson B. (2020). Effect of temperature on fusarium solani and *f. Tricinctum* growth and disease development in soybean. Can. J. Plant Pathol. 42, 527–537. doi: 10.1080/07060661.2020.1745893

[B94] Yang H. Li J. Xiao Y. Gu Y. Liu H. Liang Y.. (2017). An integrated insight into the relationship between soil microbial community and tobacco bacterial wilt disease. Front. Microbiol. 8. doi: 10.3389/fmicb.2017.02179 PMC5681905 29163453

[B95] Yang Y. Ye C. Zhang W. Zhu X. Li H. Yang D.. (2023). Elucidating the impact of biochar with different carbon/nitrogen ratios on soil biochemical properties and rhizosphere bacterial communities of flue-cured tobacco plants. Front. Plant Sci. 14. doi: 10.3389/fpls.2023.1250669 PMC10543665 37790782

[B96] Yang W. Jing X. Guan Y. Zhai C. Wang T. Shi D.. (2019). Response of fungal communities and co-occurrence network patterns to compost amendment in black soil of northeast china. Front. Microbiol. 10. doi: 10.3389/fmicb.2019.01562 PMC6629936 31354663

[B97] Yin C. Vargas J. M. C. Schlatter D. C. Hagerty C. H. Hulbert S. H. Paulitz T. C.. (2021). Rhizosphere community selection reveals bacteria associated with reduced root disease. Microbiome 9 (1), 86. doi: 10.1186/s40168-020-00997-5 33836842 PMC8035742

[B98] Zhang S. Liu X. Zhou L. Deng L. Zhao W. Liu Y.. (2022). Alleviating soil acidification could increase disease suppression of bacterial wilt by recruiting potentially beneficial rhizobacteria. Microbiol. Spectr. 10 (2), e0233321. doi: 10.1128/spectrum.02333-21 35254141 PMC9045175

[B99] Zhang T. Wang N. Liu H. Zhang Y. Yu L. (2016). Soil ph is a key determinant of soil fungal community composition in the ny-alesund region, svalbard (high arctic). Front. Microbiol. 7. doi: 10.3389/fmicb.2016.00227 PMC4767930 26955371

[B100] Zhong Y. Xun W. Wang X. Tian S. Zhang Y. Li D. (2022). Root-secreted bitter triterpene modulates the rhizosphere microbiota to improve plant fitness. Nat. Plants 8, 887. doi: 10.1038/s41477-022-01201-2 35915145

[B101] Zhou Z. Song X. Gong W. (2005). Novel conformational states of peptide deformylase from pathogenic bacterium leptospira interrogans: implications for population shift*. J. Biol. Chem. 280, 42391–42396. doi: 10.1074/jbc.M506370200 16239225

