# Supplementary material for: Metagenomic insights into the response of soil microbial communities to pathogenic Ralstonia solanacearum
Source: Front Plant Sci. 2024 Feb 16;15:1325141. doi: 10.3389/fpls.2024.1325141 (PMC10904623; doi:10.3389/fpls.2024.1325141)
Supplement: Supplementary file 2 [file DataSheet_1.pdf]

Supplementary Information

Metagenomic insights into the Response of Soil Microbial Communities to Pathogenic Ralstonia solanacearum

Yansong Xiao [a], Sai Zhang [a], Hongguang Li [a], Kai Teng [b], Shaolong Wu [c], Yongbin Liu [a], Fahui Yu [a], Zhihong He [a], Lijuan Li [a], Liangzhi Li [d*], Delong Meng [d], Huaqun Yin [d] and Yujie Wang [d *]

[a] *Chenzhou Tobacco Company of Hunan Province*

[b] *Xiangxi Tobacco Co Hunan Prov, Jishou 416099, Peoples R China*

[c] *Hunan Tobacco Research Institute, Changsha, China*

[d] *School of Minerals Processing and Bioengineering, Central South University, Changsha, China*

[*] Corresponding author: Liangzhi Li & Yujie Wang

E-mail address: 205601006@csu.edu.cn, yujie_W@163.com

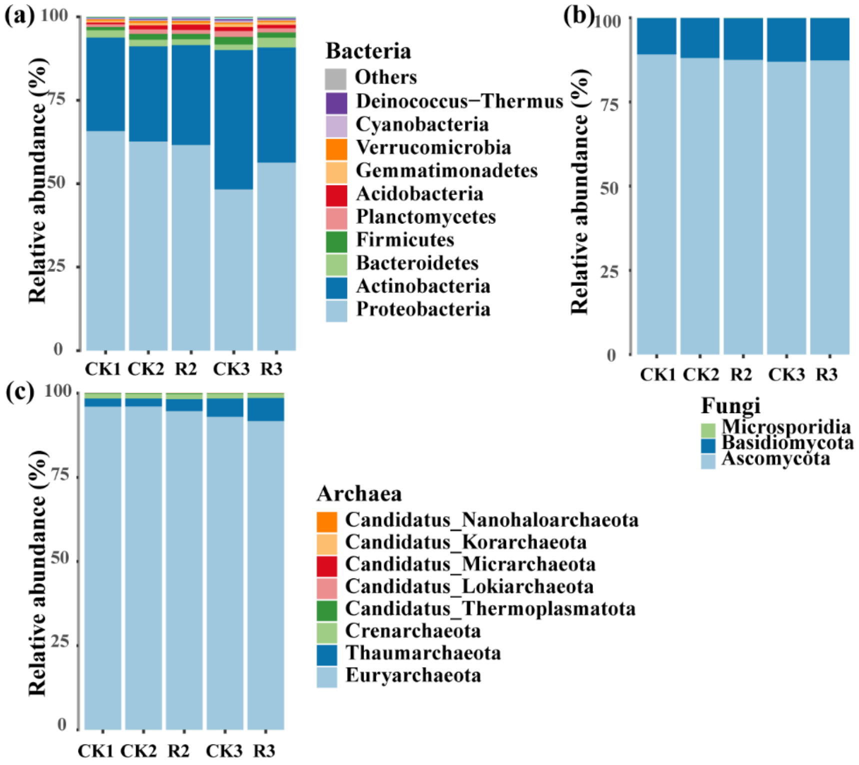

Fig S1. The relative abundance of bacteria (a), fungi (b) and archaea (c) at the level of phylum under different groups.

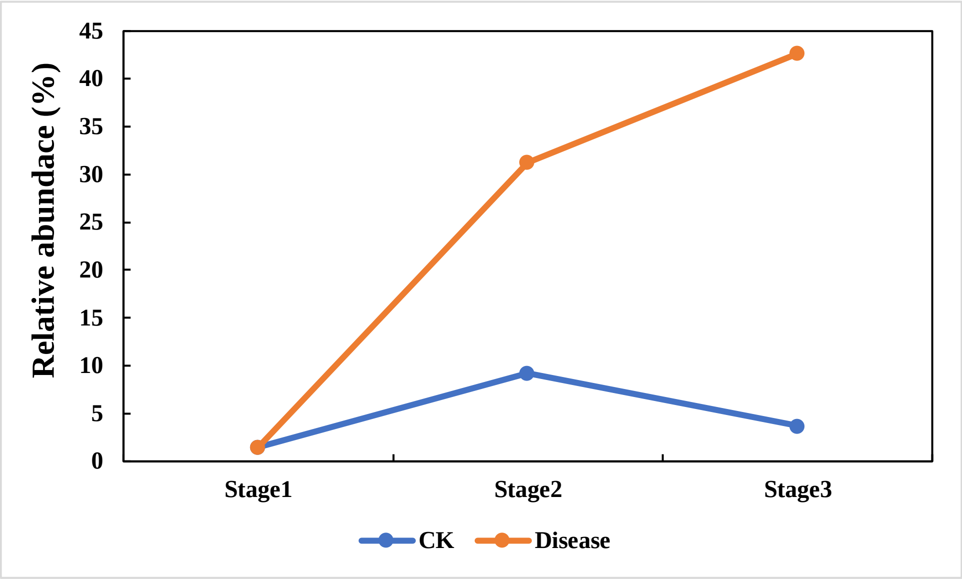

Fig S2. The relative abundance with (Disease) or without (CK) pathogenic bacteria *Ralstonia solanacearum* in rhizosphere soil under different stages.

Table S1. Taxonomy of the microbial taxa identified (using LEfSe) as key indicators discriminating of different groups.

Taxa	Group	LDA	p value
p__Proteobacteria	CK1	4.96	0.03
p__Proteobacteria;g__Ralstonia	R2	4.26	0.04
p__Proteobacteria;g__Sphingobium	CK1	4.23	0.04
p__Proteobacteria;g__Sphingobium;s__Ssp._Cam5-1	CK1	3.49	0.02
p__Proteobacteria;g__Rhodanobacter	R2	4.10	0.04
p__Proteobacteria;g__Rhodanobacter;s__thiooxydans	R2	3.43	0.03
p__Proteobacteria;g__Novosphingobium	CK1	4.04	0.02
p__Proteobacteria;g__Novosphingobium;s__resinovorum	CK1	3.32	0.02
p__Proteobacteria;g__Novosphingobium;s__aromaticivorans	CK1	3.80	0.02
p__Proteobacteria;g__Sphingopyxis	CK1	3.99	0.04
p__Proteobacteria;g__Sphingopyxis;s__macrogoltabida	CK1	3.08	0.03
p__Proteobacteria;g__Massilia	CK1	3.90	0.02
p__Proteobacteria;g__Delftia	R2	3.66	0.02
p__Proteobacteria;g__Delftia;s__acidovorans	R2	3.39	0.04
p__Proteobacteria;g__Agrobacterium	CK1	3.64	0.04
p__Proteobacteria;g__Agrobacterium;s__tumefaciens	CK1	3.30	0.04
p__Proteobacteria;g__Mesorhizobium	R2	3.56	0.04
p__Proteobacteria;g__Nitrobacter	CK1	3.42	0.03
p__Proteobacteria;g__Nitrobacter;s__hamburgensis	CK1	3.27	0.03
p__Proteobacteria;g__Ensifer	CK1	3.39	0.05
p__Proteobacteria;g__Ensifer;s__adhaerens	CK1	3.27	0.04
p__Proteobacteria;g__Bordetella	R2	3.36	0.04
p__Proteobacteria;g__Devosia	CK1	3.32	0.03
p__Proteobacteria;g__Phenylobacterium	CK1	3.09	0.03
p__Proteobacteria;g__Rhizorhabdus	CK1	3.06	0.04
p__Proteobacteria;g__Nitrosospira	CK1	3.05	0.02
p__Proteobacteria;g__Archangium	CK3	3.02	0.02
p__Proteobacteria;g__Pseudomonas;s__fluorescens	CK1	3.74	0.04
p__Proteobacteria;g__Acidovorax;s__sp._KKS102	CK1	3.19	0.03
p__Proteobacteria;g__Bosea;s__sp._ANAM02	CK1	3.17	0.02
p__Proteobacteria;g__Ramlibacter;s__tataouinensis	CK1	3.16	0.05
p__Proteobacteria;g__Variovorax;s__boronicumulans	CK1	3.13	0.03
p__Proteobacteria;g__Rhodoplanes;s__sp._Z2-YC6860	R2	3.05	0.03
p__Proteobacteria;g__Rhodoferax;s__Rsediminis	R3	3.04	0.04
p__Proteobacteria;g__Burkholderia;s__multivorans	CK2	3.02	0.02
p__Actinobacteria	CK3	4.86	0.04
p__Actinobacteria;g__Streptomyces	CK3	4.54	0.02
p__Actinobacteria;g__Pseudarthrobacter	CK1	3.73	0.05
p__Actinobacteria;g__Mycobacterium	CK3	3.61	0.02
p__Actinobacteria;g__Mycolicibacterium	CK3	3.60	0.02
p__Actinobacteria;g__Rhodococcus	CK3	3.39	0.02

p__Actinobacteria;g__Nocardia	CK3	3.36	0.02
p__Actinobacteria;g__Pseudonocardia	CK3	3.31	0.04
p__Actinobacteria;g__Actinomadura	CK3	3.18	0.03
p__Actinobacteria;g__Gordonia	CK3	3.14	0.04
p__Actinobacteria;g__Nonomuraea	CK3	3.05	0.03
p__Actinobacteria;g__Phycicoccus	CK3	3.01	0.04
p__Actinobacteria;g__Arthrobacter;s__sp._PGP41	CK1	3.30	0.04
p__Actinobacteria;g__Conexibacter;s__woesei	CK3	3.09	0.03
p__Actinobacteria;g__Nocardioides;s__sp._S5	CK1	3.08	0.05
p__Actinobacteria;g__Conexibacter;s__sp._SYSU_D00693	CK3	3.06	0.03
p__Actinobacteria;g__Amycolatopsis;s__japonica	R3	3.04	0.05
p__Firmicutes	CK3	3.81	0.02
p__Firmicutes;g__Paenibacillus	CK3	3.17	0.03
p__Acidobacteria	R2	3.75	0.04
p__Acidobacteria;g__Granulicella	R2	3.34	0.02
p__Acidobacteria;g__Terriglobus	R2	3.09	0.03
p__Acidobacteria;g__Edaphobacter	R2	3.05	0.03
p__Planctomycetes	CK3	3.67	0.04

Table S2 Topological properties of the rhizosphere soil community networks in different groups.

Network Indexes	CK	R
Number of nodes	275	278
Number of edges	3197	2746
Avg. number of neighbors	23.251	19.755
Network diameter	6	5
Network radius	4	4
Characteristic path length	2.841	2.911
Clustering coefficient	0.516	0.497
Density	0.085	0.071
Heterogeneity	0.495	0.398
Centralization	0.117	0.070
Modular	20	12
Maximal degree	55	39
Nodes with max degree	g_Cryptococcus s_gattii VGI	g_Aspergillus s_puulaauensis
